# Selection and Verification of Standardized Reference Genes of *Angelica dahurica* under Various Abiotic Stresses by Real-Time Quantitative PCR

**DOI:** 10.3390/genes15010079

**Published:** 2024-01-07

**Authors:** Jing Zhang, Xinyi He, Jun Zhou, Zhuang Dong, Han Yu, Qi Tang, Lei Yuan, Siqing Peng, Xiaohong Zhong, Yuedong He

**Affiliations:** 1 College of Horticulture, Hunan Agricultural University, Changsha 410128, China; zhangjing0711@yeah.net (J.Z.); hxinyi2023@yeah.net (X.H.); zjun2023@yeah.net (J.Z.); dzlebron0701@163.com (Z.D.); yhan2023@yeah.net (H.Y.); tangqi@hunau.edu.cn (Q.T.); yuanlei1009@163.com (L.Y.); 13272061261@163.com (S.P.); 2 College of Bioscience and Biotechnology, Hunan Agricultural University, Changsha 410128, China

**Keywords:** *Angelica dahurica*, expression stability, real-time quantitative PCR, reference genes, normalization

## Abstract

In traditional Chinese medicine, *Angelica dahurica* is a valuable herb with numerous therapeutic applications for a range of ailments. There have not yet been any articles on the methodical assessment and choice of the best reference genes for *A. dahurica* gene expression studies. Real-time quantitative PCR (RT-qPCR) is widely employed as the predominant method for investigating gene expression. In order to ensure the precise determination of target gene expression outcomes in RT-qPCR analysis, it is imperative to employ stable reference genes. In this study, a total of 11 candidate reference genes including SAND family protein (*SAND*), polypyrimidine tract-binding protein (*PTBP*), glyceraldehyde-3-phosphate dehydrogenase (*GAPDH*), actin (*ACT*), TIP41-like protein (*TIP41*), cyclophilin 2 (*CYP2*), elongation factor 1 α (*EF1α*), ubiquitin-protein ligase 9 (*UBC9*), tubulin β-6 (*TUB6*), thioredoxin-like protein YLS8 (*YLS8*), and tubulin-α (*TUBA*) were selected from the transcriptome of *A. dahurica*. Subsequently, three statistical algorithms (geNorm, NormFinder, and BestKeeper) were employed to assess the stability of their expression patterns across seven distinct stimulus treatments. The outcomes obtained from these analyses were subsequently amalgamated into a comprehensive ranking using RefFinder. Additionally, one target gene, phenylalanine ammonia-lyase (*PAL*), was used to confirm the effectiveness of the selected reference genes. According to the findings of this study, the two most stable reference genes for normalizing the expression of genes in *A. dahurica* are *TIP41* and *UBC9*. Overall, our research has determined the appropriate reference genes for RT-qPCR in *A. dahurica* and provides a crucial foundation for gene screening and identifying genes associated with the biosynthesis of active ingredients in *A. dahurica*.

## 1. Introduction

*A. dahurica* (Fisch. ex Hoffm.) Benth. & Hook. f. ex Franch. & Sav. is a perennial herb of the genus *Angelica* in the Apiaceae family, which has spread extensively throughout North, East, and Southeast Asia [1]. Numerous phytochemical studies of on this particular herb have revealed a variety of bioactive components, such as coumarins, volatile oils, glycosides, alkaloids, etc., with coumarins being the predominant compounds [1]. Latest pharmacological research has demonstrated that these components possess antibacterial, antioxidant, nerve-protection, and analgesic effects [2,3,4]. These chemical substances are often in low concentrations in plants since they are secondary metabolites produced during healthy growth or under stress conditions [5]. In order to augment their production, it is imperative to first elucidate the biosynthesis pathways involved and conduct a comprehensive profiling of gene expression pertaining to the synthesis of secondary metabolites [6,7]. This will help to provide a deeper understanding of gene functions [8]. In order to identify the transcription level of genes and improve our understanding of functional gene expression profiles, notably for coumarin production, its metabolic pathways, and regulatory mechanisms under various abiotic stresses, the identification of appropriate reference genes is urgently required.

Real-time quantitative PCR (RT-qPCR), which possesses high sensitivity, a simple operating system, and a broad range of mRNA abundance, has been extensively employed in the investigation of gene expression analysis [9]. However, several factors can impact the accuracy and reliability of RT-qPCR, such as RNA quality, the reverse transcription efficiency of cDNA, and PCR amplification efficiency [10,11]. The use of a suitable reference gene can reduce these possible inaccuracies, enabling the normalization of quantitative data across various samples. Ranking candidate reference gene expression stability under particular stresses and selecting the best candidate as the reference gene is the most dependable approach for selecting reference genes [12].

Typically, reference genes are chosen for their involvement in fundamental biochemical metabolism or the maintenance of the cytoskeleton include elongation factor 1 α (*EF1α*) [13], α-tubulin (*TUB*) [14], actin (*ACT*) [15], and glyceraldehyde 3-phosphate dehydrogenase (*GAPDH*) [16], and they are frequently selected as reference genes. However, these conventional reference genes are not always stably expressed under different conditions [17,18,19,20]. Therefore, it is essential to select suitable reference genes that maintain a steady level of expression under specific conditions. To make up for the deficiencies in traditional reference genes, some new reference genes have gradually been discovered and identified as candidate genes for standardization, such as TIP41-like protein (*TIP41*) [17], thioredoxin-like protein *YLS8* (*YLS8*) [21], and SAND family protein (*SAND*) [22], among others. Nevertheless, a systematic evaluation of reference gene selection in *A. dahurica* under abiotic stress remains unreported, underscoring the necessity of identifying and validating stable reference genes in *A. dahurica* to facilitate the accurate normalization of target gene expression.

Moreover, previous studies have indicated that plants subjected to abiotic stress exhibit increased yields of secondary metabolites, accompanied by changes in the expression levels of relevant biosynthetic genes [23,24,25]. It has been demonstrated that the application of methyl jasmonate (MeJA) leads to an augmentation in the synthesis and accumulation of tanshinones and phenolic acids within the hairy roots of *Salvia miltiorrhiza*. This effect is achieved through the upregulation of genes associated with ketone and phenolic acid biosynthesis [24]. After UV-C treatment, the physiological and biochemical parameters of cherry plants were found to have undergone significant changes, with the most notable impact on gene expression occurring in the phenylalanine metabolic pathway. This treatment stimulated the accumulation of flavonoids, anthocyanins, phenolic acids, and other related substances, thereby enhancing the antioxidant activity and nutritional value of the cherries [25]. Presently, there exists a dearth of research pertaining to the selection and evaluation of suitable reference genes in *A. dahurica* under abiotic stress conditions.

The use of unverified reference genes may lead to completely wrong conclusions. In previous studies, it has been common practice to select functional genes associated with the biosynthesis pathway for expression analysis and verification, thereby ensuring the precision of experimental outcomes [7,11,13]. Within the phenylpropane metabolism pathway, phenylalanine ammonia-lyase (*PAL*) serves as the principal and rate-limiting enzyme responsible for governing the anabolic processes involved in the synthesis of coumarin, flavonoids, and alkaloids [26,27,28]. As a result, the target gene is *AdPAL*, whose expression provides evidence for the validity of the selected reference gene.

In this study, 11 reference genes were selected based on the transcriptome data of *A. dahurica* to investigate the candidate genes most suitable for gene standardization. To comprehensively assess the stability of these genes, various external experimental treatments were applied to the plants. The original RT-qPCR data was analyzed using three statistical algorithms: geNorm, NormFinder, and BestKeeper. The comprehensive stability of these reference genes was sorted by RefFinder. To verify the suitability of the chosen candidate genes and ensure the integrity of the results, the most stable and unstable reference genes found in this study were used to measure the relative expression levels of *AdPAL* utilizing RT-qPCR. In summary, the findings lay the foundation for future investigations into the expression patterns and regulatory mechanisms of the gene for coumarin biosynthesis under various abiotic stresses.

## 2. Materials and Methods

### 2.1. Plant Materials and Stress Treatments

The experimental plants were potted and cultivated in the greenhouse of the medicinal botanical garden at Hunan Agricultural University (HUNAU). One-year-old *A. dahurica* plants were selected as the experimental subjects. For different hormone treatments, 100 μM methyl jasmonate (MeJA), 100 μM salicylic acid (SA), 100 μM abscisic acid (ABA), and 100 μM ethylene (ETH) were used to treat the plants’ leaves. Drought stress was simulated by treating the plant leaves with 100 μM mannitol. Salt stress was induced by treating plant leaves with 100 μM sodium chloride (NaCl). For heavy metal stress, 100 μM copper sulfate (CuSO_4_) was applied to the plants. A sterile water-treated plant was used as a control. Afterward, the leaves were sampled separately at 24 h for subsequent expression analysis. Prior to use, all samples were promptly filtered with double distilled water, frozen in liquid nitrogen, and placed in an −80 °C freezer. Each group of samples included three biological replicates.

### 2.2. Total RNA Isolation and cDNA Synthesis

The Prep Pure Kit (Tiangen Biotech, Beijing, China) was used to extract RNA from approximately 100 mg of the various frozen samples. The determination of both the quality and purity of the extracted total RNA was conducted using Micro-volume spectrophotometer (Shanghai BIO-DL Science Instrument Co., Ltd., Shanghai, China), while the integrity of the RNA was confirmed through electrophoresis on a 1% agarose gel. For the next experiment, only RNA samples with A260/280 ratio of 1.8 to 2.2 and A260/230 ratio higher than 2.0 were used. Then, in order to dispose of contamination genomic DNA, the RNA samples (300 ng) were processed with RNase-free DNase I (U) (Takara, Dalian, China). For qPCR, the HiScript Q RT SuperMix (Vazyme, Nanjing, China) was utilized to generate first-strand cDNA using oligo (dT) as a primer. The final cDNA samples were diluted by a factor of five using RNase-free water, resulting in a final volume of 100 μL. These diluted samples were subsequently stored at −20 °C.

### 2.3. Selection of Candidate Reference Genes and Primer Design

The transcriptome data of *A. dahurica* were used to determine a total of 11 reference genes (NCBI accession number: SRP289220). Using the BioEdit Sequence Alignment Editor (v7.0.9) program, potential single genes were screened and selected through local BLAST (TBLASTN). Matching homologues of these reference genes were obtained from Arabidopsis Information Resource (TAIR) database (http://www.arabidopsis.org, accessed on 25 September 2023). Single genes were chosen for additional examination based on their higher scores and lower E values. The Beacon Design tool (v7.9.1) was employed to design primers for each gene, with specific characteristics such as an amplicon length between 90 and 200 bp, GC content ranging from 40% to 60%, primer length of 18 to 24 bp, temperature difference between forward and reverse primers less than 1 °C, and melting temperature (Tm) between 40 °C and 55 °C. To determine optimal primer pair combination, all primers were tested by standard PCR, and the products were examined using 1.0% agarose gel electrophoresis. Furthermore, the standard curves was determined with five distinct cDNA dilutions. The formula for calculating the PCR amplification efficiency (E) was E = [10^(−1/slope)^ − 1] × 100% [29]. Table 1 provides a list of all gene-specific primer pairs designed for the RT-qPCR analysis.

### 2.4. RT-qPCR Conditions

The RT-qPCR analysis was carried out utilizing a SYBR Green Premix Pro Taq HS qPCR Kit (High Rox Plus) (Accurate Biotechnology (Hunan) Co., Ltd., Changsha, China) on a 96-well plate with an ABI 7300 real-time PCR system (Applied Biosystems). Each reaction’s final volume was 10 μL with the following ingredients: 0.5 μL diluted cDNA template, 5 μL 2X SYBR Green Pro Taq HS Premix (High Rox Plus), 0.5 μL forward primer (2.5 μM), 0.5 μL reverse primer (2.5 μM), and 3.5 μL ddH_2_O (double distilled H_2_O). The reaction was conducted under the following conditions: 95 °C for 3 min, followed by 40 cycles of denaturation at 95 °C for 10 sec, and annealing/extension at 60 °C for 30 s. The melting curve was obtained by melting the amplified template from 65 to 95 °C whilst increasing the temperature by 0.5 °C per cycle. Each RT-qPCR analysis included three technical replicates.

### 2.5. Stability Evaluation of Candidate Reference Genes

The average Ct values from three biological replicates were utilized for data analysis, and all results are shown as the mean ± standard error of the mean (SEM). Consequently, the *t*-test was used to conduct a statistical analysis. The stability of candidate reference genes was evaluated using geNorm (ver.3.5) [30], NormFinder (ver.0.953) [31], and BestKeeper (ver.1.0) [32] according to the manufacturer’s instructions. To assess the relative expression level for each gene in geNorm and NormFinder, the mean Ct value of three biological replicates was obtained using the formula 2^−ΔCt^ (ΔCt being the Ct value of each sample minus the lowest Ct value) [33]. BestKeeper directly utilized the mean Ct values in the analysis. Ultimately, a thorough stability ranking analysis was carried out with the aid of RefFinder (https://www.heartcure.com.au/reffinder/, accessed on 25 September 2023) [34,35].

### 2.6. Validation of Reference Gene Stability

To identify the stability of the selected reference genes, the expression level of *AdPAL*, which is involved in coumarin biosynthesis in *A. dahurica* [36], was determined using RT-qPCR analysis. The expression patterns of *AdPAL* in samples of *A. dahurica* under various abiotic stresses were normalized using the two most (alone or in combination) and the least stable reference genes as recommended by RefFinder. The 2^−ΔΔCT^ method, widely employed for assessing relative changes in gene expression, was utilized to compute target gene relative expression data [37]. Three technical duplicates of each biological material were used for analysis.

## 3. Results

### 3.1. Selection of Candidate Reference Genes, Evaluation of Amplification Specificity, and PCR Efficiency

Based on the transcriptome data of *A. dahurica*, we carefully selected 11 genes as potential reference genes. Table S1 shows the sequence information of the 11 potential reference genes. Table 1 presents the gene symbol, gene name, forward and reverse primers, melting temperature (Tm), amplicon size, RT-qPCR efficiency (E), and correlation coefficient (R^2^). Subsequently, the specificity of the RT-qPCR primers was confirmed through the use of gel electrophoresis and melting curve analyses. The analysis of 1% agarose gel electrophoresis revealed the presence of a single amplicon, aligning with the anticipated fragment size, following PCR amplification of all potential reference genes (Figure S1). Furthermore, the melting curve analysis exhibited distinct amplification peaks for all primer sets (Figure S2). The RT-qPCR efficiency ranged from 94% to 109%, and the correlation coefficients (R^2^) ranged from 0.9886 to 0.9973, as determined by generating a series of standard curves through cDNA dilution amplification (Table 1).

### 3.2. Expression Profile of Candidate Reference Genes

**Figure 1:**
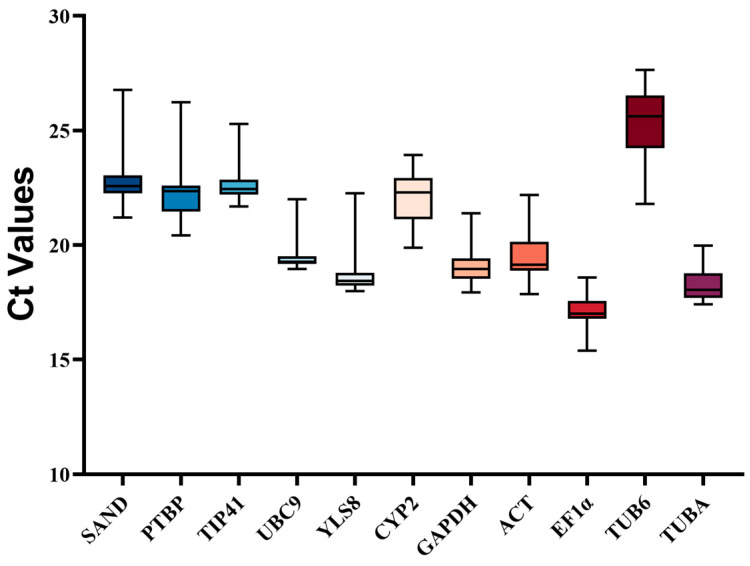
Expression profile of the 11 candidate reference genes. The box graph displays the quartile spacing. Each color only represents a different candidate internal reference gene. The inner frame of the box reflects the average value of the data, while the outer frame represents the 25th to 75th percentile. The horizontal lines below and above, respectively, showed the minimum and maximum values. The median is the line through the center of the box.

The cycle threshold values (Ct) were employed to measure the number of cycles required for the generated fluorescent signal to reach a detectable level. On the basis of these Ct values, the expression profile of the potential reference genes was consequently computed. The Ct values of these 11 reference genes are distributed between 15 and 25 (Figure 1 and Table S2). Notably, among these genes, *EF1α* exhibited the highest expression level due to it possessing the lowest Ct value. However, given the intricacy of their surroundings, it is imperative to carefully assess the appropriate utilization of these reference genes in different experimental conditions. Hence, the use of other statistical software (geNorm (ver.3.5), NormFinder (ver.0.953), and BestKeeper (ver.1.0) utilization and additional analyses are warranted.

#### 3.2.1. GeNorm Analysis

**Figure 2:**
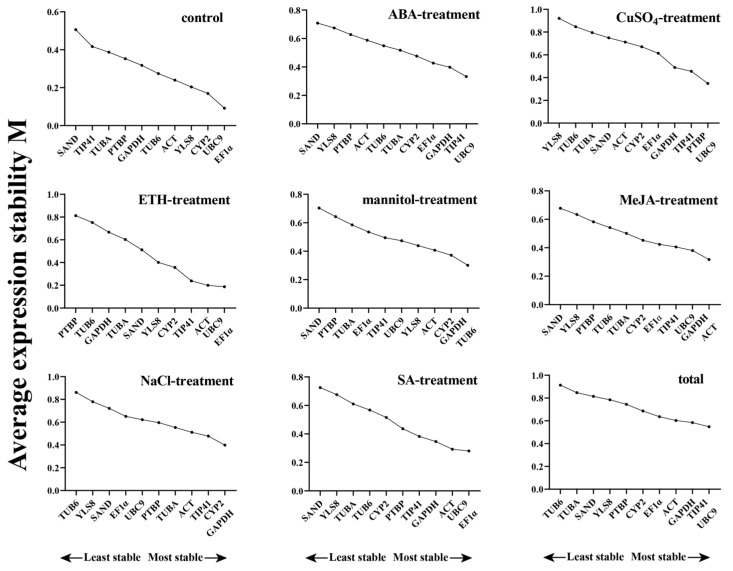
GeNorm was used to analyze the expression stability values (M) of the 11 candidate reference genes. A lower M value denotes a more stable statement. Leftward-pointing genes are less stable than rightward-pointing genes.

GeNorm analysis utilized the M value, which indicates the stability detection value of internal reference genes, to assess the stability of 11 potential reference genes. By calculating the M value of each reference gene’s stability, the geNorm algorithm filtered out those with higher stability. The general principle is that a smaller M value denotes higher stability, while a larger M value suggests lower stability. As illustrated in Figure 2, the 11 possible reference genes displayed varying degrees of stability across different treatments. *UBC9* and *TIP41* consistently exhibited the lowest M values among most treatment groups, making them the most stable reference genes. Furthermore, when considering all abiotic stress samples together, *UBC9* and *TIP41* demonstrated the lowest M values, further confirming their superior stability. Additionally, *EF1α* showed good stability under the control, ETH, and SA treatment, while *GAPDH* ranked first in the mannitol, MeJA, and NaCl treatment. *PTBP*, *TUBA*, and *GAPDH* were also among the most stable reference genes in CuSO_4_, ETH, and mannitol treatments, respectively. *SAND* generally exhibited inferior stability compared to the other assumed reference genes. Overall, *TUB6* emerged as the least stable candidate gene in our comprehensive analysis (Figure 2).

**Figure 3:**
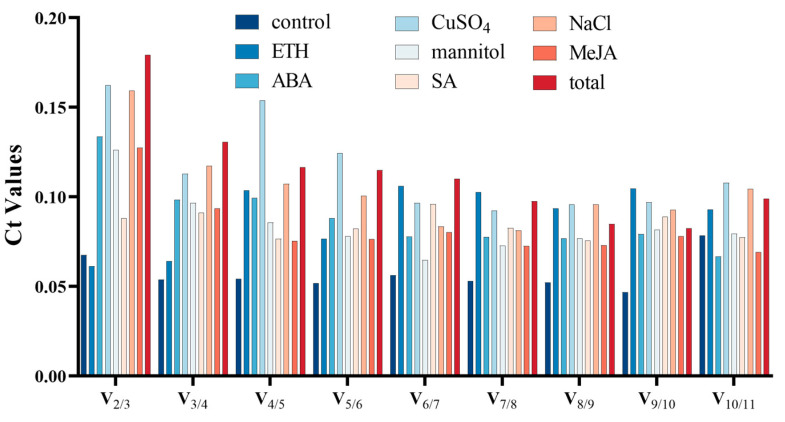
The ideal number of reference genes (V_n/n+1_) for pairwise variation normalization is determined. Columns with varying colors indicate different treatments.

The algorithm also possesses the capability to determine the paired variation V value of the standardization factor after the addition of a new reference gene. This allows for the determination of the ideal number of reference genes using the V_n/n+1_ value. A paired variation (V) score of less than 0.15 is considered ideal, suggesting that the addition of an extra gene will have minimal impact on the normalization process. The paired variation values (V_2/3_) in the control, ETH, ABA, mannitol, SA, and MeJA treatment groups were all less than 0.15, indicating that adding a third or even fourth reference gene had no discernible effect on the standardization of the target gene (Figure 3 and Table S3). As a result, the optimal number of reference genes determined under these treatments was two. Furthermore, the paired variation value (V_3/4_) of the CuSO_4_ and NaCl treatment groups is also less than 0.15, indicating that three reference genes is the ideal number for these groups (Figure 3 and Table S3).

#### 3.2.2. NormFinder Analysis

**Figure 4:**
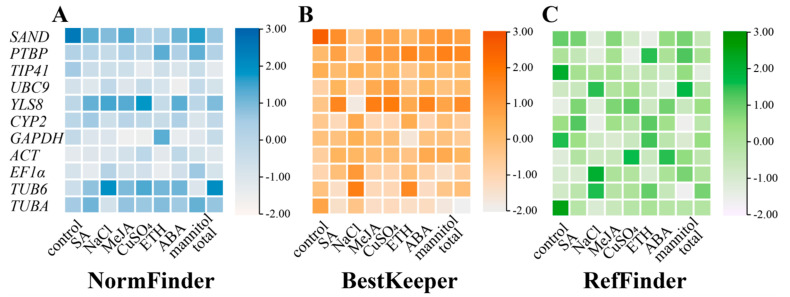
NormFinder (**A**), BestKeeper (**B**), and RefFinder (**C**) stability analyses of 11 potential reference genes. The stability of the reference gene is shown by the color change, which increases with the decrease of stability. The results of NormFinder, BestKeeper and RefFinder are listed in Tables S4, S5 and S6, respectively.

The most suitable reference gene is selected in the NormFinder analysis based on the stability value of reference gene expression, which is similar to the geNorm algorithm. The NormFinder algorithm requires that a reference gene be judged most acceptable if its expression stability value is the lowest. This algorithm can assess the variation across sample groups and compare the expression differences of reference genes. The ranks of 11 candidate genes as determined by the NormFinder algorithm are presented in Figure 4A and Table S4. In the CuSO_4_, mannitol, and MeJA treatment groups, *GAPDH* ranked first; *ACT* ranked first in the control and NaCl treatment groups; *EF1α* ranked first in the ETH and SA treatment groups. Additionally, *TIP41* consistently achieved the top ranking when considering all the sample data. Similar to the geNorm results, *SAND* exhibited instability in most cases, while *TUB6* was consistently recognized as the most unstable gene (Figure 4A and Table S4).

#### 3.2.3. BestKeeper Analysis

BestKeeper, utilizes the raw Ct values to assess the coefficient of variation (CV) and standard deviation (SD) of the reference gene from each treatment. These metrics are then used to assess and compare the stability of the reference genes. It is important to note that lower SD and Ct values indicate a higher stability of gene expression, especially when the SD exceeds 1, indicating the instability of the reference gene and rendering it unsuitable for standardization. The results are depicted in Figure 4B and Table S5, and show some variations in the ranking of each gene compared to geNorm and NormFinder. The BestKeeper algorithm determined that *TUBA* was the most stable reference gene since it consistently performed well in all treatment groups, taking the top spot in five of them (CuSO_4_, mannitol, SA, MeJA, total), thus being recognized as the most stable internal reference gene based on the BestKeeper algorithm. *YLS8* was identified as the most stable reference gene in the NaCl treatment group, while *GAPDH* demonstrated the highest stability in the ETH treatment group. *TUB6* emerged as the most stable reference gene in the ABA treatment group, and *EF1α* was deemed the most stable reference gene in the control group. The BestKeeper algorithm revealed that *YLS8* was the most unstable in most cases, whereas *PTBP* represented the most unstable gene when considering all samples together (Figure 4B and Table S5).

#### 3.2.4. RefFinder Analysis

In order to reduce the impact of constraints linked to a singular algorithm, the RefFinder application (https://www.heartcure.com.au/reffinder/, accessed on 25 September 2023) was utilized to ascertain comprehensive stability rankings for the possible reference genes. This algorithm combines the results from general analysis programs such as geNorm, NormFinder, and BestKeeper to calculate the overall ranking of 11 candidate internal reference genes. Figure 4C and Table S6 diaplayed the comprehensive ranking that RefFinder determined. Among the four treatment groups (NaCl, MeJA, CuSO_4_, and mannitol), *GAPDH* exhibited the highest stability and achieved higher rankings in most treatment groups (SA, NaCl, MeJA, CuSO4, ETH, ABA, mannitol, and total). In the SA, ETH, and control groups, *EF1α* was identified as the most stable gene. In the ABA treatment group, *UBC9* was regarded as the most stable reference gene. Considering all the sample data, *TIP41* was identified as the most stable internal reference gene. Conversely, *SAND* and *YLS8* were consistently recognized as the most unstable reference genes (Figure 4C and Table S6).

#### 3.2.5. Comprehensive Analysis

The most stable reference genes, as determined by geNorm, were *UBC9*, *TIP41*, and *GAPDH*. NormFinder identified *TIP41*, *ACT*, and *EF1α* as the most stable reference genes, while BestKeeper selected *TUBA*, *CYP2*, and *EF1α* as the most stable reference genes. According to this study, *SAND* and *YLS8* are considered the most unstable reference genes, as they consistently exhibited instability across multiple treatment groups and different algorithm analyses (Table 2). To sum up, the 11 candidate reference genes can be arranged in descending order of stability: *TIP41*, *UBC9*, *ACT*, *EF1α*, *GAPDH*, *CYP2*, *TUBA*, *PTBP*, *SAND*, *TUB6*, and *YLS8*. This result is almost consistent with the analysis results of geNorm and NormFinder, and slightly different from the analysis results of BestKeeper.

### 3.3. Reference Gene Validation

**Figure 5:**
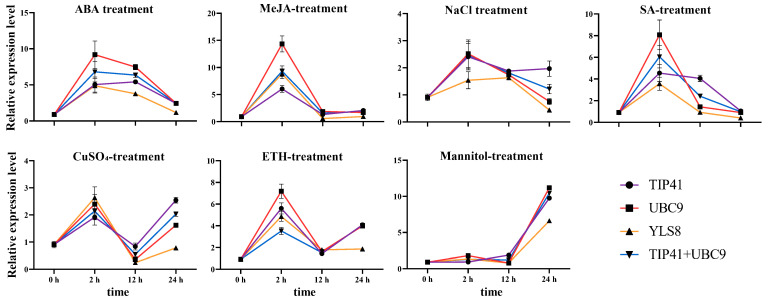
Relative expression of *AdPAL* at 0, 2, 12, and 24 h following treatment using the chosen reference genes for normalization. The most unstable reference gene (*YLS8*) in the various treatment groups and the two most stable reference genes (*TIP41* and *UBC9*) (alone or in combination) in the various treatment groups were the verified reference gene(s) used as normalization factors. Error bars displayed the standard error derived from three biological replicates.

To validate the findings obtained from the aforementioned analyses, the expression pattern of *AdPAL* was examined in all samples. The relative expression levels of *AdPAL* were calculated and utilized to assess and standardize the observed results under various abiotic stresses, thereby confirming the suitability of the selected reference gene (Figure 5). RefFinder was used to rank the two most stable genes (*TIP41* and *UBC9* under different treatments) as well as the most unstable reference gene (*YLS8*) during the validation process. The expression level of *AdPAL* in sterile water-treated leaves was assumed to be “1”, and the 2^−ΔΔCT^ method was employed to calculate its relative expression level in other samples. Figure 5 demonstrates that all samples exhibited significant changes and that the change patterns among the various sample groups altered when the relative expression level of *AdPAL* was normalized using the combination of the two most stable reference genes. During the mannitol treatment, the expression of *AdPAL* fluctuated at a low level over time and reached its peak after 24 h. In the other treatment groups, the relative expression level of *AdPAL* peaked at 2 h, with a subsequent decrease observed in the ABA, NaCl, and SA treatment groups. Conversely, in the ETH, MeJA, and CuSO_4_ treatment groups, the relative expression level initially decreased and then increased after 2 h of treatment. *AdPAL* expression patterns were consistent across all samples when normalized using the two most stable reference genes (*TIP41* and *UBC9*) alone or in combination. Nevertheless, using unstable reference genes (*YLS8*) resulted in notable variations in *AdPAL* expression patterns. In the ABA, ETH, mannitol, and CuSO_4_ treatment groups, the relative expression of *AdPAL* exhibited notable disparities. In the remaining treatment groups, the expression levels of *AdPAL* showed minimal variations, but similar expression patterns were observed when standardized using the two most stable reference genes (alone or in combination).

## 4. Discussion

*A. dahurica*, a renowned edible medicinal herb with a long history of use in China, has been found to contain coumarins as its main active component. Coumarins possess notable anti-inflammatory, antibacterial, and antitumor properties [1]. These active substances are usually secondary metabolites produced during normal growth or in reaction to stress. Previous reports have highlighted a strong relationship between the expression levels of genes associated with secondary metabolite biosynthesis pathways and the synthesis and accumulation of secondary metabolites [6,38]. The biosynthetic pathway will be much easier to understand if these genes are quantified using stable reference genes [7].

RT-qPCR remains a widely utilized method for analyzing gene expression level since its sensitivity, accuracy, and efficiency, despite the availability of advanced technologies such as microarrays and high-throughput sequencing [39]. RT-qPCR serves as a primary approach for assessing gene expression levels and unraveling plant response mechanisms under various stresses. The choice of a suitable reference gene is crucial as it lays the foundation for understanding biosynthesis and regulatory mechanisms [10,40]. Numerous suitable reference genes have been discovered in both animals and plants [14,41,42,43].

From the transcriptome data of *A. dahurica*, a total of 11 reference genes were chosen in order to assess the stability of expression in leaves when exposed to salt (NaCl), drought (mannitol), heavy metal (CuSO_4_), and hormone treatments (ETH, ABA, SA, and MeJA). The Ct values of these genes were determined for the entire sample pool (Table S2). As shown in Figures S1 and S2, the dissolution curve displayed a single peak due to the specificity of the 11 primer pairs. The candidate genes exhibited a range of amplification efficiencies from 94.53 to 109.07%, with an R^2^ value of >0.98. This gave a solid foundation for the identification and assessment of reference genes in *A. dahurica*. The mean Ct values of the 11 potential reference genes varied from 15.39 to 27.63 (Figure 1 and Table S2). Moreover, a narrow distribution range typically indicates low variability; *TIP41*, *UBC9*, and *YLS8* should therefore be regarded as stable reference genes (Figure 1). However, since the expression level of reference genes could vary under different experimental conditions (Table S2), it is essential to analyze their stability separately for specific tissues and treatments, utilizing multiple tools.

In this study, the stability of putative reference genes was sassessed using three statistical algorithms: geNorm [30], NormFinder [31], and BestKeeper [32]. Finally, the results were combined and verified using RefFinder. Previous studies had emphasized the necessity of using two or more algorithms to ensure accurate results. With a cut-off value of 0.15 (V_n/n+1_), geNorm was able to ascertain the ideal number of reference genes for normalization. In this study, the V_2/3_ values for leaves under drought and hormone treatments (ABA, SA, ETH, MeJA) were below 0.15, suggesting that two reference genes would suffice under these treatments (Figure 3 and Table S3). According to this rule, three genes should be used for leaves treated with CuSO_4_ and NaCl. However, the 0.15 threshold was not an absolute requirement. Moreover, given that the V_2/3_ values for the CuSO_4_ and NaCl groups were slightly below 0.15 (0.012 and 0.009, respectively) (Table S3), using three reference genes instead of two during reference gene validation may not be necessary [41]. Similar to geNorm, NormFinder selected the most suitable reference gene by analyzing the relative expression levels of the reference gene. In the SA, MeJA, ETH, ABA, and mannitol treatment groups, the most stable reference genes were determined by the NormFinder algorithm, and the overall sample data were consistent with the results obtained from geNorm (Figure 4A and Table S4). However, the most stable genes identified by NormFinder in the NaCl and CuSO_4_ treatment groups differed from the geNorm results, with *ACT* and *GAPDH* ranked as the most stable genes in these two groups. The ranking of the most unstable internal reference genes was mostly consistent across different analyses, except for the ETH and ABA treatment groups.

Notably, while geNorm and NormFinder produced similar rankings for the reference genes, there were significant variations in the outcomes obtained from BestKeeper (Table 2). BestKeeper often identified low-ranked internal reference genes as the most stable genes according to the first two algorithms (excluding the control group) (Figure 4B and Table S5). In summary, the disparities between geNorm and NormFinder suggested that the ranking order of reference genes may vary based on the specific experimental conditions. The discrepancies observed between geNorm, NormFinder, and BestKeeper indicate that the ranking order may differ among the analysis method (Table 2). These variations can be attributed to the differences in the procedures and statistical algorithms employed. Previous studies have also reported instances where the results obtained from BestKeeper were inconsistent with those of geNorm and NormFinder, as observed in *Nitraria tangutorum* [43] and *Kentucky bluegrass* [42].

Finally, RefFinder, a comprehensive tool, was used to generate the final rankings of the 11 candidate genes by analyzing the geometric mean of rankings from each algorithm. A lower geometric mean indicates greater stability of the gene (Figure 4C and Table S6). The reference gene selected for standardizing and quantifying the expression level of *A. dahurica* was determined by RefFinder, with *UBC9* and *ACT* following *TIP41* as the most stable reference genes. These results agreed with previous studies conducted on *Momordica charantia* [22] and *Psoralea corylifolia* [21], where *TIP41* also demonstrated high stability. However, in studies on *Angelica decursiva* [17] and *Dendrobium huoshanense* [44], *TIP41* was not identified as the most stable gene, further highlighting the absence of a universal reference gene that is stably expressed across different conditions or organisms. Hence, it is essential to verify presumed reference genes before each RT-qPCR analysis to ensure their suitability for specific experimental conditions. Furthermore, previous studies have indicated that the most stable internal reference genes are not always stably expressed in various species or under specific experimental conditions [17,21,22,44]. Therefore, when selecting suitable reference genes, it is vital to consider the differences in species and experimental conditions.

To further assess the suitability of the selected reference genes, the expression of *AdPAL*, a crucial enzyme for the synthesis of coumarins in *A. dahurica*, was normalized using the two most stable genes (*TIP41* and *UBC9*), their combination, and the least stable gene (*YLS8*) under various treatments. Coumarins represent the primary active components of *A. dahurica*, and the expression level of *AdPAL* may directly correlate with the coumarin content, which could provide a basis for further research on biosynthesis regulation [27]. The results demonstrated that significant variations occurred when the most unstable reference gene, *YLS8*, was used for standardization (Figure 5). When one stable gene or a combination of stable reference genes was used for normalization, slight variations were observed in the expression pattern of *PAL* (Figure 5). These findings underscore the significance of selecting appropriate reference genes to ensure accurate RT-qPCR results, as exemplified by the expression analysis of *PAL*. Notably, in the different treatment groups, the expression of *AdPAL* exhibited a pronounced increase after 2 h of treatment. This could be attributed to the enhanced formation of coumarins, a type of secondary metabolite in *A. dahurica*, following stress treatments, consequently resulting in an increased relative expression level of the *AdPAL*. Similar observations have been reported in cherry [25] and potato [45]. Interestingly, during continuous treatment, only the expression of *AdPAL* in the ETH, mannitol, and CuSO_4_ treatment groups showed an upward trend, while the other treatment groups exhibited a decline.

## 5. Conclusions

In summary, this study aimed to identify stable reference genes in *A. dahurica* under various treatments. Of the 11 candidate genes investigated, *TIP41* exhibited the highest stability, followed by *UBC9* and *ACT*. Conversely, *YLS8* was found to have low stability and is generally not advised as an appropriate reference gene in most circumstances. Furthermore, the results were validated by assessing the relative abundance of *AdPAL*. Overall, this research contributes to our understanding of gene expression in *A. dahurica* under several abiotic stresses and can be an effective tool in the selection of relevant reference genes in other plants.

## Figures and Tables

**Table 1:** Candidate genes and primer pairs used for qPCR normalization in *A. dahurica*.

Gene Symbol	Gene Name	Forward/Reverse Primer (5′-3′)	Tm (°C)	Size (bp)	E (%)	R^2^
*SAND*	SAND family protein	GCTTGCCTTGTTGGATAA	59.4	101	100.40	0.9964
		GATGCTGTGTTCTCTTCTC	59.4			
*PTBP*	Polypyrimidine tract-binding protein	GTGTGATTGGAACTTGATG	58.1	149	94.53	0.9886
		TTACTGATGCTGAGACTG	57.4			
*GAPDH*	Glyceraldehyde-3-phosphate dehydrogenase	GTATCCGTTGTTGACCTT	58.4	120	100.12	0.9961
		ATCACAGACCTCCAGAAT	58.6			
*ACT*	Actin	TTCACCATTCCAGTTCCA	59.9	147	107.35	0.9902
		TATCCGCTTCATCGTTACA	60.1			
*TIP41*	TIP41-like protein	GCAAGTGAGAGGTCTGAA	60.4	184	106.82	0.9857
		ACAAGTCGGAATATGGAAGT	60.5			
*CYP2*	Cyclophilin 2	AACAGCGACATAATCAAGC	60.1	125	105.68	0.9901
		CTCTCCAGCACCAAGTAA	60.0			
*EF1α*	Elongation factor 1 α	GATTGGAGGTATTGGAACT	58.0	158	107.59	0.9973
		CATTGTCACCAGGAAGAG	58.8			
*UBC9*	Ubiquitin-protein ligase 9	CCTCCAGATAGTCCTTATTC	57.9	165	109.07	0.9973
		GCTCCATTGTTCTTTCAG	57.4			
*TUB6*	Tubulin β-6	TGAGATAAGGAAGAGTAGTG	57.1	92	102.06	0.9911
		ACATAGGCATAAAGGGATAC	58.2			
*YLS8*	Thioredoxin-like protein YLS8	CTGTTGCGGAGACGATAA	60.9	191	100.67	0.9953
		CTGCTTGTCCTTCATTGC	60.5			
*TUBA*	(Tubulin-α)	AAGGTATGGACGAGATGG	59.6	181	101.41	0.9974
		TCAGCAGTTCACACAGTA	59.6			
*PAL*	Phenylalanine ammonia lyase	GGTGATGGAGAGTATGAA	56.7	170	105.61	0.9989
		GTGGTAATGTGTTGGAAG	57.0			

**Table 2:** Rankings of the 11 candidate reference genes based on stability values calculated using different types of algorithms.

Rank	1	2	3	4	5	6	7	8	9	10	11
SA											
geNorm	*EF1α/UBC9*	/	*ACT*	*GAPDH*	*TIP41*	*PTBP*	*CYP2*	*TUB6*	*TUBA*	*YLS8*	*SAND*
NormFinder	*EF1α*	*UBC9*	*ACT*	*GAPDH*	*TIP41*	*PTBP*	*CYP2*	*TUB6*	*TUBA*	*YLS8*	*SAND*
BestKeeper	*TUBA*	*TUB6*	*CYP2*	*GAPDH*	*EF1α*	*TIP41*	*ACT*	*UBC9*	*PTBP*	*SAND*	*YLS8*
Comprehensive Ranking	*EF1α*	*UBC9*	*ACT*	*GAPDH*	*TUBA*	*TIP41*	*TUB6*	*CYP2*	*PTBP*	*YLS8*	*SAND*
NaCl											
geNorm	*GAPDH/CYP2*	/	*TIP41*	*ACT*	*TUBA*	*PTBP*	*UBC9*	*EF1α*	*SAND*	*YLS8*	*TUB6*
NormFinder	*ACT*	*GADPH*	*TUBA*	*TIP41*	*CYP2*	*PTBP*	*UBC9*	*EF1α*	*SAND*	*YLS8*	*YUB6*
BestKeeper	*YLS8*	*UBC9*	*PTBP*	*SAND*	*TUBA*	*ACT*	*GAPDH*	*TIP41*	*CYP2*	*EF1α*	*TUB6*
Comprehensive Ranking	*GAPDH*	*ACT*	*CYP2*	*TUBA*	*TIP41*	*PTBP*	*UBC9*	*YLS8*	*SAND*	*EF1α*	*TUB6*
MeJA											
geNorm	*ACT/GAPDH*	/	*UBC9*	*TIP41*	*EF1α*	*CYP2*	*TUBA*	*TUB6*	*PTBP*	*YLS8*	*SAND*
NormFinder	*GAPDH*	*UBC9*	*ACT*	*EF1α*	*TIP41*	*CYP2*	*PTBP*	*TUBA*	*TUB6*	*YLS8*	*SAND*
BestKeeper	*TUBA*	*TUB6*	*CYP2*	*EF1α*	*GAPDH*	*ACT*	*TIP41*	*UBC9*	*SAND*	*PTBP*	*YLS8*
Comprehensive Ranking	*GAPDH*	*ACT*	*UBC9*	*EF1α*	*TUBA*	*CYP2*	*TIP41*	*TUB6*	*PTBP*	*YLS8*	*SAND*
CuSO_4_											
geNorm	*UBC9/PTBP*	/	*TIP41*	*GAPDH*	*EF1α*	*CYP2*	*ACT*	*SAND*	*TUBA*	*TUB6*	*YLS8*
NormFinder	*GAPDH*	*TIP41*	*EF1α*	*UBC9*	*CYP2*	*ACT*	*PTBP*	*SAND*	*TUBA*	*TUB6*	*YLS8*
BestKeeper	*TUBA*	*TUB6*	*CYP2*	*EF1α*	*GAPDH*	*ACT*	*TIP41*	*SAND*	*UBC9*	*PTBP*	*YLS8*
Comprehensive Ranking	*GAPDH*	*TIP41*	*UBC9*	*EF1α*	*PTBP*	*CYP2*	*TUBA*	*ACT*	*TUB6*	*SAND*	*YLS8*
ETH											
geNorm	*EF1α/UBC9*	/	*ACT*	*TIP41*	*CYP2*	*YLS8*	*SAND*	*TUBA*	*GAPDH*	*TUB6*	*PTBP*
NormFinder	*EF1α*	*ACT*	*UBC9*	*TIP41*	*CYP2*	*YLS8*	*SAND*	*TUBA*	*TUB6*	*PTBP*	*GAPDH*
BestKeeper	*GAPDH*	*TUBA*	*TIP41*	*UBC9*	*ACT*	*EF1α*	*SAND*	*CYP2*	*YLS8*	*TUB6*	*PTBP*
Comprehensive Ranking	*EF1α*	*UBC9*	*ACT*	*TIP41*	*GAPDH*	*CYP2*	*TUBA*	*YLS8*	*SAND*	*TUB6*	*PTBP*
ABA											
geNorm	*UBC9/TIP41*	/	*GAPDH*	*EF1α*	*CYP2*	*TUBA*	*TUB6*	*ACT*	*PTBP*	*YLS8*	*SAND*
NormFinder	*UBC9*	*GAPDH*	*TIP41*	*EF1α*	*ACT*	*PTBP*	*CYP2*	*TUBA*	*TUB6*	*SAND*	*YLS8*
BestKeeper	*TUB6*	*TUBA*	*CYP2*	*EF1α*	*GAPDH*	*UBC9*	*TIP41*	*ACT*	*SAND*	*PTBP*	*YLS8*
Comprehensive Ranking	*UBC9*	*GAPDH*	*TIP41*	*EF1α*	*TUB6*	*CYP2*	*TUBA*	*ACT*	*PTBP*	*SAND*	*YLS8*
mannitol											
geNorm	*TUB6/GAPDH*	/	*CYP2*	*ACT*	*YLS8*	*UBC9*	*TIP41*	*EF1α*	*TUBA*	*PTBP*	*SAND*
NormFinder	*GAPDH*	*TUB6*	*CYP2*	*ACT*	*TIP41*	*UBC9*	*YLS8*	*EF1α*	*TUBA*	*PTBP*	*SAND*
BestKeeper	*TUBA*	*EF1α*	*TUB6*	*UBC9*	*GAPDH*	*CYP2*	*TIP41*	*ACT*	*YLS8*	*SAND*	*PTBP*
Comprehensive Ranking	*GAPDH*	*TUB6*	*CYP2*	*ACT*	*TUBA*	*UBC9*	*EF1α*	*TIP41*	*YLS8*	*PTBP*	*SAND*
control											
geNorm	*EF1α/UBC9*	/	*CYP2*	*YLS8*	*ACT*	*TUB6*	*GAPDH*	*PTBP*	*TUBA*	*TIP41*	*SAND*
NormFinder	*ACT*	*EF1α*	*UBC9*	*TUB6*	*GAPDH*	*YLS8*	*CYYP2*	*PTBP*	*TUBA*	*TIP41*	*SAND*
BestKeeper	*EF1α*	*UBC9*	*ACT*	*CYP2*	*YLS8*	*TUB6*	*GAPDH*	*PTBP*	*TIP41*	*TUBA*	*SAND*
Comprehensive Ranking	*EF1α*	*ACT*	*UBC9*	*TUB6*	*CYP2*	*YLS8*	*GAPDH*	*PTBP*	*TUBA*	*TIP41*	*SAND*
Total											
geNorm	*UBC9/TIP41*	/	*GAPDH*	*ACT*	*EF1α*	*CYP2*	*PTBP*	*YLS8*	*SAND*	*TUBA*	*TUB6*
NormFinder	*TIP41*	*ACT*	*EF1α*	*UBC9*	*GAPDH*	*CYP2*	*PTBP*	*SAND*	*TUBA*	*YLS8*	*TUB6*
BestKeeper	*TUBA*	*CYP2*	*EF1α*	*GAPDH*	*TUB6*	*UBC9*	*TIP41*	*ACT*	*SAND*	*YLS8*	*PTBP*
Comprehensive Ranking	*TIP41*	*UBC9*	*ACT*	*EF1α*	*GAPDH*	*CYP2*	*TUBA*	*PTBP*	*SAND*	*TUB6*	*YLS8*

## Data Availability

The gene sequence information mentioned in this study can be found from the literature based on the gene list in Additional Table S1. The raw data can be accessed from the NCBI Sequence Read Archive (SRA) platform under the accession number SRP289220. The materials are available from the corresponding author on reasonable request after publication of the work.

## Supplementary Materials

The following supporting information can be downloaded at: https://www.mdpi.com/article/10.3390/genes15010079/s1, Figure S1: Agarose gel (1.0%) electrophoresis showing amplification of a specific PCR product of the expected size for each gene. Figure S2: Melting curves of 11 candidate reference genes tested in this study. Table S1: Information of all candidate genes and target genes in *A. dahurica*. Table S2: Ct values of 11 candidate genes of *A. dahurica*. Table S3: Pairwise variation (V_n/n+1_) analysis of 11 candidate reference genes calculated using geNorm. Table S4: Stability rank of 11 candidate genes by NormFinder. Table S5: Stability rank of 11 candidate genes by Bestkeeper. Table S6: Expression stability values of 11 candidate genes calculated using the RefFinder.
