# Supplementary material for: Selection and Verification of Standardized Reference Genes of Angelica dahurica under Various Abiotic Stresses by Real-Time Quantitative PCR
Source: Genes (Basel). 2024 Jan 7;15(1):79. doi: 10.3390/genes15010079 (PMC10815136; doi:10.3390/genes15010079)
Supplement: Supplementary file 1 [file genes-15-00079-s001.zip › genes-2796965-supplementary.pdf]

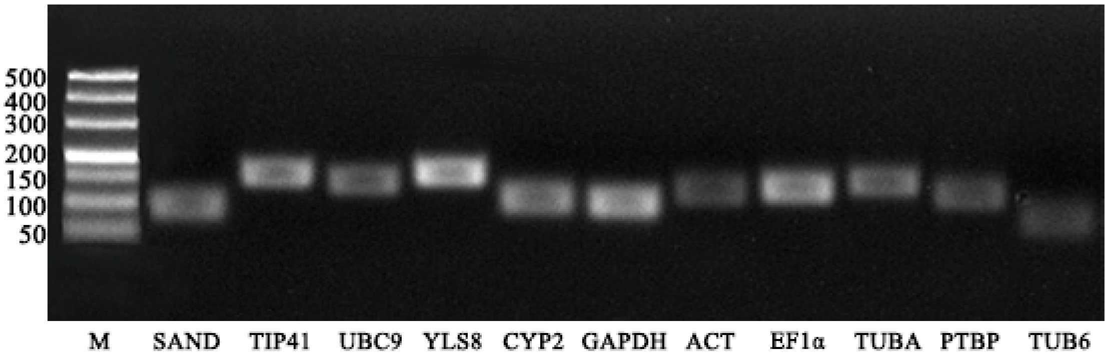

Figure S1. Agarose gel (1.0 %) electrophoresis showing amplification of a specific PCR product of the expected size for each gene. M represents a 500 bp DNA marker.

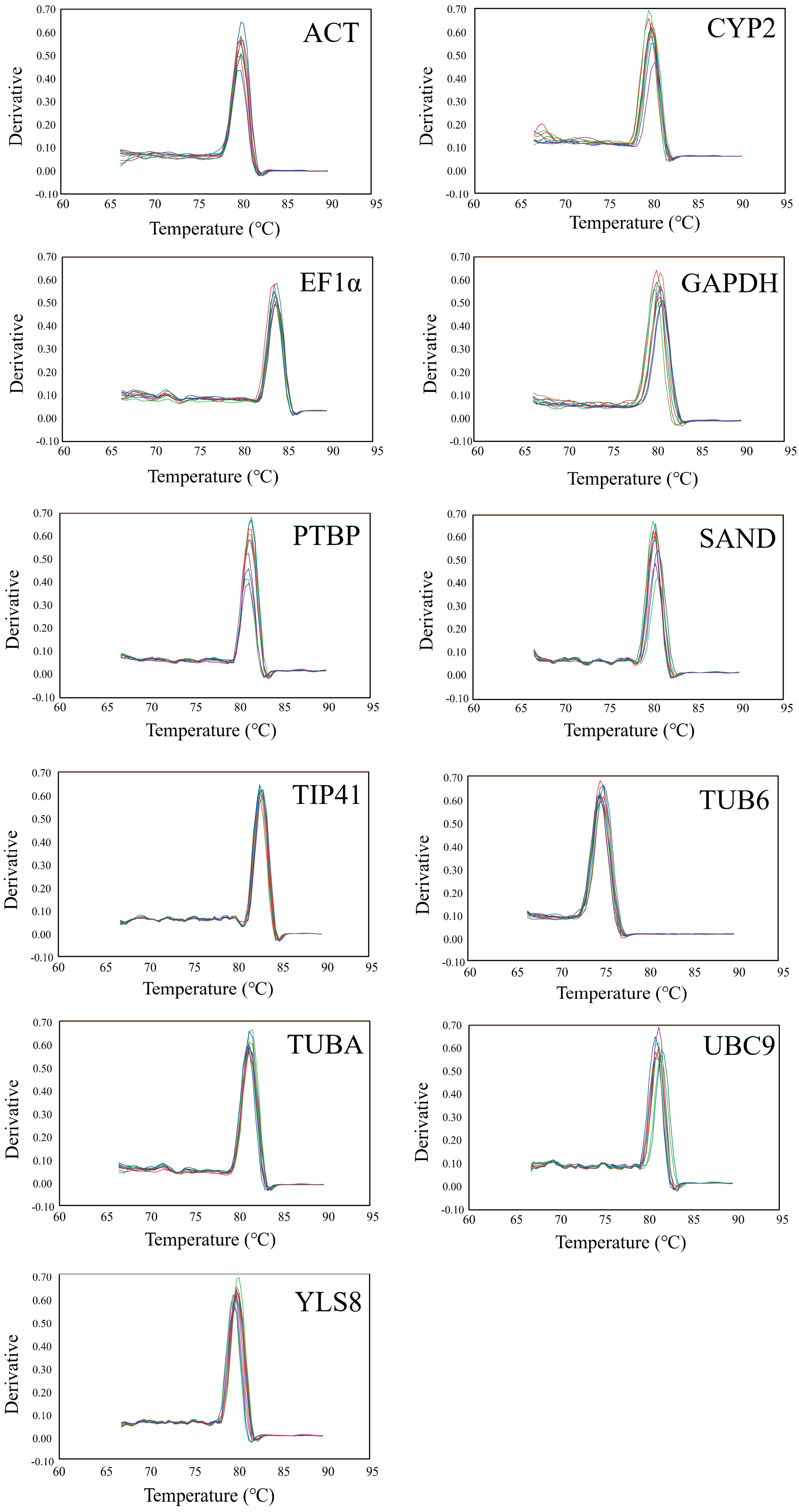

Figure S2. Melting curves of 11 candidate reference genes tested in this study.

Table S1. Information of all candidate genes and target gene in *A. dahurica*.

Gene abbreviation- *SAND*. **Gene ID-** TRINITY_DN30497_c0_g1_i3
Gene sequence-
CTGGCGGTACATTAACAATTCAGGTGCTTCTATTGCAATATTGCAAAATTGTAGGTTGGCCTCAATGAA
ATATACCTAGAGATGGATTACAGACTTAGCATACAAACATACAGTAAATGACAAATACATCTGATGA
TACATTCAACGAACTAATAGGATTGATATATTATGATCGGCATAATCTTGCTGTGTTAGAACCTTGTTC
AAAAATCAAAACTAGTATACATGAATAACATACTTTTCTGGAAACATCACCATGAAAAGGGGCTTGCT
CCCAACAAAAAAACTTCATTTTCCACATCTTTAACCCATTGGCATACTCGGTTGCATGTCTTTATAGCC
AGTGCCTTGTCTGCTAGAGGATCAAATGCTGCATAAAGTTCAAAATCCTGAGTAACCCAGCAGAGTAT
AACATAGTTCTCATCCCTTCTAAACTGGGTTTTGTGAGGACCAAGTTCTCTATCATGCATAGAAGTATA
CAGCTTCTGATATGCTCTATATAATCTTTTCTGTTGTTTAGGGGTGTTGATCGGTGATGAGAACTCAGAA
GATACATATTGATCTAGATACATACTGCGGTAAACGAAGTGCCAAAGCCCAGCAGGACCACCTAACA
GTGCCTTACACCTATCTGCGGAGTCCAGTGGAGGTCTAGGCTGACCTAAATGAGATGACAAAGATCCC
GAGCGAGGAGATGGATTAACAGGCAAATCTTCGACACGCATGCCACCATCCAACATCGACCTTTGAG
CTTCATTAAGAACATTTGACTTCAGAAGGACCATTTCGATACGGATCCTCCAATCTTTTAGACGATGAA
ATGCATCAGGATTAGCAGTAAGCAACATCAAATATGTATCAGCATCAAGATAATACACATAACTATAT
AAAAATGCCATTGGATTGTATCTCGGCAGACAGATTGGAGAGAAAGATTCAGATGTCCTGAATGATTC
AGATGACATCACAAAGTTGGCAAGCAAAAGCATATCATCAGGATGAAGAGATGCTTTTTGTGCACCA
ACCAGACTGATGACCTTGTGTTTACACATTAATATCGCGAAGAGGACACCTGACTCAGCAACATCCTG
CAATATGGCACCGGCGGCTTGCCTTGTTGGATAAGCAAGGGGAAGACAAGAGTAGGCATGAAGAAA
AGTGGCAGGGTTCCAACTAAACGAGTGGATGAGAGAAGAGAACACAGCATCTGTTCCCCCAAGCAA
AGGCATCATATCAAATTTCGGATTCTTCTCAAAGCATCTATTTATAGACTTTGTCAGAATAAGTATCAT
CTGGCCATAAAGAAGTTCCAGTTGTTCACTGAGGGATTCATGAGGCTCTTCTGTACAGCTTATGCAAAC
TAGATATATAGGTCCTTTAACAAGAAAAACCACCTGGTGTTTTCCCGCCCTAACCAACTTCACACGAT
CTCCCCCATTCTCCACGAAGGAAATGATGGCTTGCAAAGTTGCTGAAAATCCTGCTAGTCTGTGTTCAT
CTCCATATCTTGAATATATTGGTTTTCCGGAATGGCTTAAGATAAAAAAATGCTTCTTCCTTTTCCTCCA
TGAAGCAGAAGCATCATCTTCATTAACATGCCATTTCCCAGGGACCCATTCCGAATCCACACTACAAC
CAAAAGAACCACTCCTGTTATCCACCTCATCATCGCCACTAATCTCCTCGATACCTGAACCACTTATAC
CACTACTACTACTAGTCCCCTTTCCTCCAGCATATCCACTACTACTCGGACTCCCTTGCCCTTCCACCTC
AACTTCCACATCCGAATTATTCCTCCACAACACTCCTTCGCCTCTCGAATTTCCAACTACTTCCTTCTCT
ACTTCTGTATACGAATCCGAACCTAATATATCCACTTCTCCAGAATTTTCAACTACTTCTCCGACTCGC
GAATGTGAATCTGAAAGTAATGTACCGGTATTTTCTGGATTTTCAGCTACTGCTACTTGTTCGAGTTGC
AAATGTGAATCAGAAGGTAATGTATCGATAGTTTGGGGATTCTCAGCTACTTCGAGTTGCGAATGTGA
ATCGGAAGGTAATGTATCGATATTTTCGAGATTTTCAGCTACTACTTCGAGTTGCGAATGCGAATCGGA
AGGTAATGTAGCGGCATTTTGGAGATGAGGGGGTATTAGGGGTTTTGAGTTGTGGTGATTGAGTGAAA
TAGAGGTTAATTGACCTTCAATAGCGTCGAGAGATTGGTCAATAGAATCATGAATACAAGCTGAATTA
GGAGTAGCAGATGATGACCCTGCAATGCTAGTAGATAAGCTCCCAAGTTCTGTTCCAGTCTCATTGTGT
ACAATTTCCTTTGAATCAGAAGATACAGAGTCTGAATTAGCTACTGCTGCGATTCCGATATCGATCGCC
GTTGAATTCTCCATTTTGATCGAAATTTTAGCACTTATAAACAGAGTTGTGTATATATACACTTATACA
GGAAGGAGAGAGAGAGGGGGGGGGAGAGAGTATAAAACAATCAGAGAAGAGAGAGAGAGTTATAA
AACAAAGAGATGGTGTTATAAAATAAAGAGAGAGAAAG

Gene abbreviation- *PTBP*. **Gene ID-** TRINITY_DN27766_c3_g2_i10
Gene sequence-
GACAAAAAATAGGTACTTGGGTAAGAACCCCCCAGAATAACCAAAAAAAAAAAAAGCTTTCCAGTCA
ATGTCACTCAAACTGTGACCTAGAAACATATTTATCACGCCACTAGAATTTTAAGTAGAACACAGTAC
AATCTGCCATCCGCTACTTGATGTTTACAAGGATATGCACACATATCCTTGGTTGGACACCAAAGGAC
AGTAAATAGAACAAAAACAATTTGAAGAATTCAAGTCTACTGAACAGAACTGATACAACTTACTATC
AGTAAGGTGGGTGGTGGTGATTATGTGGGGAAGAACTATCATGTTGCATATTTGACTGATTCCCAGGC
TGACCTCCACTTGCTGAAGAACTGGGATTGATTTGGCTGCTGTGTCCAAGAGGTGAGGAACCAGCAGG
AGAGGCCATAAGATTATAAGCCTGACCAGAGGGAGGCCCATAAGTTTGTCCAAGTAATGTGTTAGGG
CCTGAAACAAAGGAGGGTCTGCTAAGCTGCATGGCTGGATTCCAAGATGGAGTTTGTCCAGGCGGGC
CCTGCATGGGAGGTCCGTGGGCAGGAGGTCCTTGCCCGGGAGGTCCGTGGTCAGGTGGACCTTGACCG
GAAGGACCGTGGCCAGGAGGTCCTTGACCGGGAGGGCCGTGGCCAGGAGGTCCTTGACCGGGAGGG
CCGTGGCCAGGAGGTCCTTGACCGGGAGGACCGTGGCCAGAAGGTCCTTGACCGGGAGGGCCTTGAG
GCTGCCCATAATTGATACTAGAAAATTCGCTCCCAATAAAGACCGGAGCAGCATGAGGATTCTGCCAT
ACTGTTGCTCCAGCAGGCAGACCAGCAGCAAAACCGGACTCTGGTACTGTATAATCCCTACTTTTATC

GCTGAAGGCCTTTACATTGAGATCAGTATGACGAGAGTATGATATATGAAGCTTACAGTAGCCACCAT
CATATATGCAGTGTCCCTCTAGAGCATCTTTAGCTACAGCGGCAGTGTTGACATCAGGATACTGAATTA
GAGCCTGAGTTGTCGCGTTCTTCTCGAATATAGCAATTTTCTGAACCGTACCAAATGCAGAGAATACC
GTGTTAAGAACATCTACAGTGACATCATAGATCCTATTTTCAATTGAGGCAAGAAGTACATTACTCTCC
GGTTCTTTTTTCTTTCCGTCAGGACCTACAACAGGCTGGACAAATCCCTCAATTGCAGTTGCATTAACA
GGAAGATATGGATTTGTATAGTCCCGGCTACGGTGTGATTGGAACTTGATGTTTAGATCTGTGTGAGCT
GAATATGAGATGCGCAGATTGCAAGAACCAACATGTTCTGGAAGCAAGTACCTTGGAATACTTCTGCC
ATCTAAAGCTTCCCTTGCTGAAAGAGCAGTCTCAGCATCAGTAAACTGGATTAGTGCCTGGAAACCTG
CTGCCTTCTCAAAAGTAGCAATCTTGTGCACAAATCCAAAAGCCGAGAAGACCAAGTGAATCACATC
AATGCTTACATCACCGGCTTCTACACCCTCAATGGTTACCAGCAAGACATTTCCCGGAACATCACCTG
GACCCTTGTTGTTGACAATTTCATGTCTGTTTGAATACTGTATATAAACATGTTTACCCCGAACAGATG
CAGGTTCCGATGATGAAGCATAATATGAAACCATATTAATGGCCTGATTAAGATCCACAAATTCAACG
AAGGCTTGATTGCGATTAGCGCCGACATTGCACTTGGTGTTAACGATCTTACCAAAAGGCTTGCAAAG
CTCAACGAGCTCTTCTTCGATACACTCCCAAGGCAAGTTACGCAAGTGAAGCACTTTAGATGGAGTCT
GTGTGTATCGAAATTGAGGCTGATTTGAATTCGACATCCGCTCACTTTTGATTCAATTAGGGTTTTGTTT
GTGTGTGTATATATATGTAGTATTATTTGTATGGGTGGTGCTGAATATGCAGAGGAGAGCTGAACGTTT
T

Gene abbreviation- *GAPDH*. **Gene ID-** TRINITY_DN26270_c0_g5_i1
Gene sequence-
CAAAAATATCATCTCTTATCTTATAGAAATCTCTACATTTATCCTCACCACACACTCAGTTTGTAACCTC
AAGATGCATATTCAGCATTAAAAACTTATATAGTTTCTCTTTTGCAAGGAACTTTTTAATAATATTTGTT
CAATTTAATAAAGACAATATCTTAATAGAAAAAAAATACAGAATAACACAAAATTAACTTGAGATGG
AACTAATGAGGAGTTCAATTAAGCTGCATGATAGAGTTGGAAGTAAAATGTAAGTGTATTATGTATAA
ACACTGAAAAATAAGAAGAAAAAAAGTAGGTATTCTCTGAGTCTTTAGTTGCAGCAATGGCTTGTAA
AGCAGCTCTGGTTTCTTCAAGCCTTCCAGTAAATATTAGACTTCCATCTAGAAGCTCTTATTCTTTCCCT
GCTCATTGCTCCTCCAAGAGACTTGAAGTAGCTGAGTTTTCCGGGCTCCGATCCAGTGTTTCGCTCACA
TATTCAAAGAATGCTAGTGAAGGATCTTTCTTTGATGTGGTGGCTTCCCAACTTTCTCCTGAGACTTCA
GGATTATCCCCTGTTAGAGGACAAACAGTTGCCAAATTGAAGGTAGCAATCAATGGTTTCGGACGCAT
TGGCAGGAATTTTCTTCGTTGCTGGCATGGACGCAAGGACTCTCCACTCGAAGTTATTGTTGTCAATGA
CAGTGGTGGTGTCAAGAATGCATCGCACTTGCTCAAGTACGACTCAATGCTGGGCACTTTCAAGGCAG
ATGTTAAGATTGTAGACAGTGAGACCATCAGTGTTGATGGAAAGCACATCAAGGTTGTCTCCAACAGA
GATCCACTCCAGCTTCCTTGGGCTGAGATGGGAATCGACATTGTTATTGAGGGTACTGGTGTGTTTGTT
GATGGCCCTGGGGCTGGGAAGCACATCCAAGCAGGGGCAAAGAAAGTTATTATCACCGCACCAGCA
AAAGGAGCTGATATTCCAACTTACGTTGTTGGCGTGAATGAGAAAGATTATGGTGATGACGTTGCTAA
CATCATAAGCAATGCTTCTTGCACTACAAACTGTTTAGCTCCTTTTGTGAAGATCTTAGATGAAGAATT
CGGTATCATTAAGGGAACCATGACAACCACTCATTCTTATACAGGAGACCAGAGGCTTTTAGATGCTT
CTCATCGGGACTTGAGGAGAGCCAGAGCTGCAGCTTTGAACATAGTCCCAACAAGTACTGGTGCAGC
CAAGGCTGTATCTTTAGTGCTACCCCAACTGAAGGGTAAGCTGAATGGCATTGCACTCCGGGTGCCCA
CGCCTAATGTATCCGTTGTTGACCTTGTTATAAATGTCGAGAAGAAAGGACTTACTGCTGAAGACGTC
AATGCTGCCTTTAGAAAGGCTGCTCAAGGACCACTGAAGGGAATTCTGGAGGTCTGTGATTTGCCTCT
AGTGTCGGTGGATTTCCGGTGCTCTGATGTTTCATGCTCCATCGATTCATCATTGACAATGGTCATGGG
GGATGACATGGTCAAGGTCGTAGCCTGGTACGACAACGAATGGGGATACAGCCAAAGAGTTGTAGAT
TTGGCAGATTTGGTAGCAAGCAAATGGCCAGGTGCAGCAGTAACTGGAAGTGGTGATCCATTAGAAG
AGTTTTGCGAGACAAACCCTGCTGATGAGGAATGCAAAGTTTATGAATAATTTACCTTTGTTCCACCTT
TATGCTTCCCAATTAAGTTGTTTAGGTGGTTGTTAAGTTCAAGTCATTCGATGTGTTAGTGCGTCATGAT
TTGTTTCGGAAGTGCAGTGATAATATGGAATCAGAGGATGGGGAGAGTCGGTCTAAACTACCAAGTG
CAGGGGAAGGTATCGGAAATATGCTGGTTCTTGCATTATTAGATTGGTGCATAATTGGGTTAATTTGAA
ATGAACTGGTGACTGAAATAAGATCTGTGGGGAACATCAAGATATTGATGTTTTCAAAGATAGATGCC
TGTCTGTTTCAAAATTATTAAGCTTTGTTTTAATTATTACTCTTGACCGAATGGCCAACATAGAGGCTGC
AACTGTTTTCTTGCTCTGCCTTTTACTGCTGTTTTTCTCTCCTTAATTAGTGGCAACATTTGATAATGCTT
AGCAGCTGTAAGAGCTCCAACTGCATAAAAGTAAACATACCATGACAATGTGCTCATACGGACTCGA
CCAAGTCAAGAAAATAGACAGCAACCTCGCTCATGTAACTGATTCCCACCTAAACCAGCTAATAGAT
AGACCTCCAAACTTACTTGTAAGCAACTTTTGTAGCAGCACCAAACACTCCAAACTTCTGAGCAGCTG
AAGTGCTATACATTACAATGGCTCATTCTTCTCATTTTTTC

Gene abbreviation- *ACT*. **Gene ID-** TRINITY_DN29396_c0_g2_i4
Gene sequence-
AATATAAACATGTGTTGCAGTAAAAGGAAAGAAAATGAAATTAAAGTATTTAATAATTGGATAGGAA
AATCAACATAACAAACTCAACTCTAAGCATCCTACTAACAAGTCACAATTTTCTTTAACATACAGACT
GATATACCAAACATAGTAGACAAAGAGTAGATGCATCAAAATCATTTTATCCTCTCTCCCAATTGTCA
ACCCATCACAGGTAACCAAGTTCACATGAAAAATTACATTAAAAGCCAAATAAATTGAAAAAGAAA
CTCCGAGTTCCAATGCATATAATACTGTAGAACTTAGAAGCACTTCCTGTGCACGATTGATGGGCCAG
ACTCATCATATTCACCCTTGGAGATCCACATCTGTTGGAAGGTGCTCAGTGATGCAAGAATGGATCCT
CCAATCCAGACACTGTATTTTCTCTCAGGTGGTGCAACAACCTTGATCTTCATGCTGCTGGGAGCAAGA
GCAGTGATTTCCTTGCTCATACGATCTGCAATACCGGGGAACATTGTCGAACCACCACTGAGCACTAT
GTTTCCATAGAGATCCTTCCTGATATCAACATCACACTTCATGATGGAGTTGTAAGTAGTTTCGTGAAT
TCCTGCAGCTTCCATTCCGATCAAAGATGGCTGGAACAGAACCTCAGGGCAACGGAATCTTTCAGCTC
CAATTGTAATAACTTGACCATCAGGCAATTCATAATTCTTTTCCACAGAAGAGCTACTCTTCGAAGTTT
CAAGTTCCTGCTCATAGTCGAGGGCAACATAAGCAAGCTTCTCCTTCATGTCACGCACAATTTCCCGCT
CGGCAGTGGTGGTGAACATGTAACCTCTCTCAGTTAAGATCTTCATGAGAGAATCAGTGAGATCACGC
CCAGCAAGATCAAGACGAAGAATGGCATGGGGAAGGGCATACCCTTCATAAATTGGTACAGTATGGC
TCACACCATCACCAGAGTCGAGCACAATACCAGTAGTACGTCCACTTGCATATAGAGAAAGGACGGC
CTGGATCGCAACATACATAGCAGGAACATTAAATGTCTCAAACATGATTTGAGTCATCTTCTCCCTGTT
GGCCTTAGGGTTGAGTGGTGCCTCAGTTAGAAGAACTGGGTGTTCCTCTGGAGCAACTCGAAGCTCAT
TGTAAAAAGTGTGATGCCATATTTTCTCCATGTCATCCCAATTACTTACAATACCATGCTCAATTGGAT
ATTTCAAGGTAAGAATACCTCTCTTCGACTGGGCTTCGTCACCAACATAAGCATCCTTCTGTCCCATCC
CAACCATCACACCAGTATGTCTTGGTCTCCCAACAATACTGGGGAACACCGCTCTGGGAGCATCATCA
CCAGCAAAACCAGCCTTCACCATTCCAGTTCCATTGTCACAAACTAGGGGCTGGATATCCTCAGCATC
GGCCATTTTATGTTACTGATAAAGAGTTGCTTTATCTCTCTCGATAATCTCTCTCTCTCGATAATCTCTCT
CTGTTGTAACGATGAAGCGGATATGAGAGTAGTG

Gene abbreviation- *TIP41*. **Gene ID-** TRINITY_DN25936_c0_g1_i2
Gene sequence-
TTAAAAGATGTATAATGCAGAAGGTGTTTACATGCCTCTGGAAATTTGTCTCAAATCTCAATTCAAGA
GCAAGGAATATGGTAATGTATGCAGCTGGATGCCAAAGTAAAGATAAAAACATTATGCAGTAAAAAG
TAAAATACTGTGAAGATATAAATAAAACTTTCCCAGCATGTAATGATAAAATACAGCATTTCCATACT
ATAAAGGATTAGTATAAGCGCACTGCGTACATGTTAATCAGGAATTATAAGCTTTTGGGTCTTATGCA
AAATGATAGGAAGTCTTTGGCTGATGCTGCTTGGATCAATATACGCAGCACAATCAGAAGGATATCCT
TTAGAAGCTAGTGCTTGAAATGTGGCTTCTCTCCAGCAACATTCTCTCAGAATAACTGGTTTTTACCCT
CACCAAAAATGCAATGGATGCGTGTGTCTCTTAAACGCATAAGCACACCATCAACTCTAAGCCAAAA
ACGCAAGAGAAGAAACCAACAGCTTGGCATCACTCTCACTTTTACTGTTAAAAGCGACACTCCACTAT
CAGCCAATTCATCTTCATAGAAGATCACCTCATCATAGAAGAGAATAGGCTCTTTTGATGCAAGTGCA
GTCAAATTAATTCGTTCCTCGCAGTCCTCCCAACGAAGCTTGCAACTGCCTTCATCAGAGATTGTATCC
CTCTCTGAGTTTTTCTCAACAGTTTCACTTCCACAATATGGTGTTGTAAATGTGTAGTCATAATCTAATA
TCACCTGCTGGAAAGGTTTGCATCTAAATTTCCAATTTGCTGCTGCAGGGACCTCAACTGGCGGTAATT
TTTCCTGCTTCCAACCAACTAGAGAATCAAATGCGTTGAAATGAATCTTTACATCGCTCTTCAAGTGCT
TGAGAACCAATGAACTTTCCCCAAAAACCATTTCTGGCAAGTGAGAGGTCTGAAGCTTTTGTTCCCAC
AATTGGACGTTGACTGAGTTGAGGATGGGGAACTTGCGAGTTTCAATCTCCCAATCGTTAATTATTAAA
CCAGTTCTTCCGTCAGGAAGGAGCTGAGCTCCGGCAGCTTTAAGCTCTTCCTTGTCGATCTCAACTTCC
ATATTCCGACTTGTTTACCTGATCAGCCGGTCACCAGTTGCCTATATATATCTTCAAGTGACAGCGAGC
TCTAATGATTCTTTTTTCCGATTTTCA

Gene abbreviation- *CYP2*. **Gene ID-** TRINITY_DN25407_c0_g2_i1
Gene sequence-
TTTAGTTAAAAAAAACACACACAAAACACATACAACTCTCTCCGATCTCTCCACCGTCGTTGATCTCTC
TCCGGCAGGCTTGAAGGTGGGAGCTAGACGGGAACTCAGGCTCGTGCCTTGTGATGCCAGGTGTAGCT
TGACAAGTGCACGTTTGTATGCTGTGTTCAGTTATAGAAAATATGGGTTCTTTCTGCAGTAAACAGCGA
CATAATCAAGCTGATTCCGAAGAAAATGCACAGACTGCAGAAATAGAAAGGCGAATTGAGCTAGAA
ACAAAGGCAGAGAAACATATACATAAGCTTCTTTTACTTGGTGCTGGAGAGTCAGGAAAATCAACAA
TCTTTAAGCAGATAAAACTTCTATTTCAGACCGGCTTTAATGAGGCTGAGCTAAAGAGCTACATTACA
GTTATTCATGCAAATGTGTATCAGATAATAAAAATATTATATGATGGGGCAAAGGAATTGGCTCAAAA
TGAAGAAGAATCCTCAAAATATGCTTTATCGGTTGAAATCAAGGAAATAGGAGAGAAACTATCGGAA
ATTGGTGGCAGGTTGGATTATCCTCGCCTCAGTAAAGAGCTTGCTCAAGAAATAGAAACTCTCTGGAA

AGACGACGCGATACAGGAAACATATTCTCGGGGTAATGAACTCCAAGTTCCTGACTGCACTCTTTATT
TCATGGAAAATTTACAAAGGTTGGCTGTTGCGGATTATATTCCGACAAAGGAGGATGTTCTTTATGCA
AGGATCCGTACAACTGGTGTTGTAGAAATTCAGTTCAGCCCGGTTGGAGAGAACAAAAAAAGTGGCG
AAGTATATAGATTATTTGATGTTGGAGGTCAGAGAAATGAGAGAAGGAAATGGATTCATCTGTTTGAA
GGTGTTACAGCTGTGATTTTTTGTGCTGCCGTTAGTGAGTATGATCAGACTCTTTTTGAGGACGAGAAC
AGGAACAGAATGATGGAGACAAGAGAACTCTTTGAGTGGGTCCTCAAGCAGCCATGTTTCGAGAAAA
CATCCTTCATGCTGTTTCTGAACAAATTTGATATATTTGAGAAGAAGGTTCTAGATGTGCCACTAAATG
TATGCGATTGGTTTAAAGATTACCAACCAGTATACTCGGGAAAGCAGGAAGTCGAGCATGCATATGA
GTTTGTCAAAAAGAAATTTGAGGAGCTATATTTTCAAAGCACTGCCCCTCACTGCGTGGACCGAGTTTT
TAAGATATATAGGACAACTGCTCTTGACCAGAAGCTTGTGAAAAAGACTTTCAAGCTGGTAGACGAG
ACCTTAAGAAGGCGAAACCTCTTCGAAGCGGGTTTGTTGTGATAGTGGATTTCATCCGAAAGGAAATG
TTAATATCTTGAAAAGCTAGATTCCTGCAGTGAGGTGGGAACACTCATTAAACTCTTTACACCGTAATT
TACAGGTAAAAAATTGGCTGTTTGGTATACCCTCTGTAATAGGAACTTGATGTTACCCCTGTTTCTTTTT
GCCTTAAAAGAAGTCCCCAAGATTTTGTTTTCTGTTTGTAGCATACCGCTAGTTGGAGAAGCAAGCAA
GTATATCAAAAGTTTTTTTATACCTTAATTTCGTTTTTGTTTTTTTACAGTGATCTGTTTTATGTTTTACCT
AAGAATTG

Gene abbreviation- *EF1α*. **Gene ID-** TRINITY_DN32140_c1_g3_i6
Gene sequence-
CAAGAATTGAGGTCAATAACAAAAAAAATACTAATATCGTCCATTCATTTAAAGATTTTTTTCCTCTAT
ATAAAGTTTCTATCAAGTTTTACAACACCAGTCTCTTCTTTCATCAAGGCCTTTTTTTGCAACATATATA
GAGTTCATCTCTTTGACAAGTTAGTAGCTGATCACAATGGGCAAGGAGAAGGTTCACATCAGCATTGT
GGTGATTGGCCATGTGGATTCCGGAAAGTCAACCACCACTGGTCATCTCATCTACAAGCTTGGCGGGA
TTGACAAGCGCGTGATTGAGAGGTTCGAGAAAGAAGCAGCTGAGATGAACAAGCGTTCGTTCAAGTA
TGCGTGGGTGCTTGACAAGCTCAAGGCTGAGCGTGAGCGTGGTATCACTATTGATATTGCTCTGTGGA
AGTTTGAGACTAACAAGTACTACTGCACTGTCATTGATGCCCCTGGACATCGTGATTTCATCAAGAAT
ATGATTACTGGAACTTCTCAGGCTGACTGTGCTGTTCTTATCATTGATTCAACTACTGGAGGTTTCGAA
GCTGGTATCTCCAAGGATGGGCAGACGCGTGAGCATGCCTTGCTTGCTTTCACACTTGGTGTCAAGCA
GATGATTTGTTGCTGCAATAAGATGGATGCCACAACTCCCAAGTACTCCAAGGGTAGGTATGAAGAA
ATTGTGAAGGAGGTTTCTTCGTATTTGAAGAAGGTTGGGTACAACCCCGACAAAATTGCATTCATTCCC
ATCTCTGGATTTGAGGGTGACAACATGATTGATAGGTCTACCAACCTTGACTGGTACAAGGGACCGAC
TCTTCTTGAAGCTCTTGACCAGATCTCTGAGCCCAAGAGACCATCAGACAAGCCCCTTCGTCTCCCACT
TCAGGATGTTTACAAGATTGGAGGTATTGGAACTGTTCCAGTGGGACGTGTTGAAACTGGTGTGATCA
AGCCTGGTATGGTTGTGACTTTTGGTCCTTCAGGGTTGACCACTGAAGTCAAGTCTGTTGAGATGCATC
ATGAGGCTCTCCAGGAGGCTCTTCCTGGTGACAATGTTGGATTCAATGTTAAGAATGTTGCTGTTAAGG
ATCTCAAGCGTGGATATGTTGCCTCCAACTCCAAGGATGATCCCGCCAAAGAGGCTGCCAACTTCACT
GCTCAAGTTATCATCATGAACCACCCTGGTCAGATCTCAAATGGTTATGCTCCAGTTCTTGATTGCCAC
ACCTGTCACATTGCTGTTAAGTTTGCTGAAATCCAAACCAAGATTGATCGTCGATCGGGTAAGGAGCT
TGAGAAGGAGCCCAAGTTTTTGAAGAATGGTGATGCTGGATTTGTTAAGATGATTCCAACCAAGCCAA
TGGTGGTTGAGACCTTTATGACCTACCCTCCTCTTGGAAGGTTTGCTGTTAGGGACATGAGGCAGACTG
TTGCTGTGGGAGTCATCAAGAGTGTGGAGAAGAAGGAACCTACCGGAGCCAAGGTCACAAAGGCGG
CAATCAAGAAGAAATGAACCGGTGCTGTTGGTGGGTATCGGTGTGGTAATACTGCAGGCTGGATTCGT
CTTGTGGTTTTAATATTAAGTCAAGTTTCTGTTATGTTTCGTGTCATGTGTTTTGCTGTGTCAGCCACCTG
GTCGCAGAATTGGGTGCTTGACAGGCGGTGGTGTGGCTATTATTAGCCTTGTTATCCTTGTGTTCGTTTT
CTTTCTTGTTTTCTTTCTTGTTTTAAAAGACTACCGTGTTATTTGATGTTATATGACAGACCAGCCGTTGA
TTTTGGTGTTTATGTTGCCCTTGAGTGTTGTGATTTCTTTTTGCCAAGTTTATATGTTTAGTTTCATCTACG
AAAGACTGCCGTTTTGTTTAGATATCTTGGACAATGTTATTTACCAGGTAAGATTGAAGTGGAAATTGT
TTCTTCAAGTGTAACAGTAACGTGGTTAGTCATCCATGTATGTATGTTTGAAACCTCCATCTCATTTCGG
AAAATTGCTTTTTTC

Gene abbreviation- *UBC9*. **Gene ID-** TRINITY_DN32451_c0_g1_i7
Gene sequence-
CACGCTACTATCATCATTTTCTCTACATCTTCCACTTCATTTCAGCTTCCATCTTTCACATTTCTTGGTAA
GTAGTTGACGATGGCTTCTAAGAGGATTTTGAAGGAACTTAAGGATCTTCAGAAGGATCCTCCTACTTC
TTGTAGCGCTGGCCCTGTTGCTGAAGATATGTTTCACTGGCAAGCTACAATTATGGGCCCTCCAGATAG
TCCTTATTCTGGTGGGGTTTTTCTCGTTACCATACATTTCCCTCCAGATTATCCGTTCAAGCCACCAAAG
GTGGCATTTAGAACAAAGGTTTTCCACCCGAATATAAACAGCAACGGGAGCATATGCCTAGACATTCT
GAAAGAACAATGGAGCCCTGCTCTGACCATTTCCAAGGTTTTGCTGTCAATATGCTCCTTGCTGACAG
ACCCAAATCCGGATGACCCTTTGGTGCCCGAGATAGCTCACATGTACAAGACAGACAGAAACAAGTA

TGAAACAACTGCAAGGAGCTGGACCCAGAAGTATGCCATGGGTTAAGAAGCTATGCGTTTATGATGG
AATTGCCTTTCTCTTAAAAATAATT

Gene abbreviation- *TUB6*. **Gene ID-** TRINITY_DN9948_c0_g1_i1
Gene sequence-
CTCTCTCTCTCTCTCTCTCTCTCTCTCTCTCTCTCTCTCTCATGTGTAGATCTACGAATGTTGGCAATGTA
AATGAACTCGTTGAAATGTGTCCATATTTTGTTTATATATATTCTGATTTTTGTATGGTATTTTGTTTTATT
ATTGTTGAAGATGAGATGACTACAGATCTTCGAGTATTGGTTATTTATATGAAGTTGTTGAAATGTTTG
TGTGTGTGTGCATGCATAGATCTGCGAGTATTGATGTGTAAATGAACTTGTTGAGATGCGTGTATATAT
GTGTGTGTATGAGATAAGGAAGAGTAGTGTGTTTACTAATTGTTTTTTTGATTATTATTCACATTACGTA
TTGCTCGAGCTGTATCCCTTTATGCCTATGTTTGTTTATTAAGTATACAAGTGTATTATGTTTTGTGTCTG
ATCTGATTCATATCTTCGTGATTTGTATGTTTTATTCAGTCTCTAGGTCACTAACTGTAAGACAGTAATG
TATATACAGATCTGTATAGATTTAAACAAGCATGTGATTCTCGAATGTTCTGTATGGGTGTCAATTAAC
TGAGCACATTCTTGTTTGTTTAGGTTTATATGCAGGTGTTTGTTCTCATGTGTTTCTTCATGATTTGGTTTT
AGAGATTTATATGATGTTGTGAGGTCTTTATTATTTTTAATTTGAGAAACTAATGCTAGACTTGATATGA
CATGTGTGTATGTATAAGATTTAGATATTGTGCTGATTATTA

Gene abbreviation- *YLS8*. **Gene ID-** TRINITY_DN29984_c0_g1_i5
Gene sequence-
GGTGAGAGAGATGAGCGAGAGAGATGAGACAGAGAGGAGAGAGTCGTTGTGTGAAAGGGGGGTGAT
TGATAGTTGATACAATCGTCTATACCTAGATATCGAGAGAGAGATCGAGAGAGATCGAGATCGAGCG
AGAGAGAGAGAGGAGAAGATGTCGTATTTGCTTCCACACTTGCACTCAGGATGGGCAGTAGACCAGG
CGATTCTCGCTGAGGAAGAACGCCTCGTCGTCATTCGCTTCGGTCACGATTGGGATGAAACTTGTATGC
AGATGGATGAAGTACTAGCTTCTGTTGCGGAGACGATAAAGAACTTTGCTGTCATTTATCTCGTGGATA
TTACTGAGGTGCCCGATTTCAACACTATGTACGAACTGTACGATCCTTCTACTGTTATGTTCTTTTTCAG
GAACAAGCATATTATGATTGATCTTGGCACTGGGAACAACAATAAGATTAATTGGGCAATGAAGGAC
AAGCAGGAGTTTATTGACATTATTGAAACGGTTTATCGTGGAGCAAGGAAAGGCCGTGGATTGGTCAT
TGCACCCAAAGATTACTCCACCAAGTACCGCTATTAGTCTGTCCTGCATCATTTGGCAGGTTGACGAG
GCTAAACAATACTTTAACTGTTGAAAAGTATTATGTGACATCAAATTGTTCAAGTGAGTTGGGCCTTAA
GGACTGTGTGCACTTTGAATGTGTGAAGGAACAAGGACATGGCAGAAATACCACTGAAGTTGCAGCG
TCAATTTGCTACGTTCCTTCAACAGGGTAGTCTCTAGCTCTACTCTTAATTTACTGTGTGCACTTTGAAT
GTGTGAAGGAACAAGGACATGGCAGAAATACCACTGAAGTTGCAGCGTCAATTTGCTACGTTCCTTCA
ACAGGGTAGTCTCTAGCTCTACTCTTAATTTACT

Gene abbreviation- *TUBA*. **Gene ID-** TRINITY_DN27287_c1_g6_i1
Gene sequence-
CTTTGGTGATCTCAACCACTTGATTTCGGCCACAATGTCTGGTGTTACATGTTGCTTGCGTTTCCCTGGT
CAGTTGAACTCTGACCTCAGGAAGTTGGCTGTAAATCTAATCCCCTTCCCTAGGCTTCACTTCTTTATG
GTGGGTTTTGCACCTCTTACATCCCGTGGTTCCCAACAGTATCGTGCTTTGAGTGTGCCTGAGCTTACCC
AACAGATGTGGGATTCCAAGAACATGATGTGTGCGGCTGATCCCCGCCATGGTAGATACTTGACAGCT
TCTGCTGTGTTCAGAGGAAAGATGAGCACCAAGGAGGTTGATGAGCAGATGATCAATGTTCAGAACA
AGAACTCATCTTACTTTGTTGAATGGATCCCAAACAATGTGAAGTCAACTGTTTGTGATATCCCACCGA
CTGGCTTGAAAATGGCTTCTACTTTCATTGGGAATTCGACTTCAATTCAGGAGATGTTCAGGCGTGTGA
GTGAGCAGTTTACTGCTATGTTCAGGAGAAAGGCCTTTTTGCATTGGTACACAGGCGAAGGTATGGAC
GAGATGGAATTCACCGAGGCTGAAAGCAATATGAACGATCTTGTTTCTGAGTATCAGCAGTACCAGG
ATGCAACTGCTGATGAGGAGGGAGAATATTTTGAAGATGAAGAAGAGGAGGGGCAAGAAATGTAAG
GTGTGTTCTGCCATGAGTGTACTGTGTGAACTGCTGAGTATGTAGGATGCTGCAACTGTGTGTTGCCTTT
TGGGCAGTCTTCTGAAGCTGTTGTCTAGCTATTATGTGGTTTGTTTAATTTCTCAAAGACAGTTTGCGA
AATCTTGTTAAGAATTTAACTTGGCTTTGTTATTATATTCAGTTTTATTTGCAGATTTAGTAGTTTTTGGT
TGCTTGGGTTACAAAAGCAAAGTTTACTTAATGATT

Gene abbreviation- *PAL*. **Gene ID-** TRINITY_DN30247_c1_g2_i1
Gene sequence-
ATGGATTGCCAGACCAAGAATGTTGTGTCAGTAAACGGGACAGGTGATTCGCTATGCATGCAGAAAG
ATCCTTTGAACTGGGGAATGGCTGCGGAGTCATTGAAAGGAAGTCATTTGGACGAAGTTAAGAGGAT
GGTGGCTGAGTTTAGGAAGCCTATGGTGCAGCTGGGTGGAGAGACATTGACGGTTTCTCAAGTGGCCG
CTATTGCTTCCGGTTACGTCAAGGTTGAGTTATCGGAGACGGCAAGGGCTGGAGTTAAAGCTAGTAGT

GATTGGGTGATGGAGAGTATGAATAAAGGAACCGATAGTTATGGTGTTACTACCGGTTTTGGTGCTAC
TTCTCATAGGAGGACTAAACAAGGTGGTGCTTTGCAGAAAGAACTTATTAGGTTTTTGAATGCTGGAA
TCTTTGGAAATGGAAGTGATTCTTCCAACACATTACCACATTCAGCAACAAGAGCAGCAATGCTTGTG
AGGATCAACACACTTCTCCAAGGTTACTCTGGCATTCGATTTGAGATCCTTGAAGCAATTACAAAGTTT
CTCAACCAAAACATTACTCCTTGTTTGCCACTCCGCGGAACAATCACTGCTTCTGGTGATCTCGTTCCC
TTATCCTACATTGCTGGACTTCTAACAGGACGTCCCAATTCTAAGGCGGTCGGACCCACTGGAGAAAA
TCTAACCGCTGCAGAAGCATTTAAACTTGCTGGAGTTGATGGTGGATTCTTTGAGCTGCAGCCAAAAG
AAGGCCTTGCTTTAGTCAATGGAACGGCTGTTGGTTCTGGGATGGCTTCGATGGTTCTCTTTGAGACCA
ATATACTTGCTGTGTTAGCTGAGGTTATGTCAGCTATATTTGCTGAAGTGATGCAGGGCAAACCAGAAT
TCACTGACCACTTGACACATAAGTTGAAGCATCATCCAGGACAAATTGAAGCCGCGGCCATCATGGA
ACACATATTGGACGGTAGCTCTTACGTTAAGGCTGCTCAGAAGCAACATGAAATGGATCCCCTGCAAA
AACCAAAACAAGATAGATATGCTCTTAGGACGTCTCCACAATGGCTTGGACCGCAGATTGAAGTAATT
CGATCATCAACAAAAATGATAGAAAGGGAGATCAATTCTGTCAATGATAACCCTTTGATTGATGTGTC
AAGGAACAAGGCCATACATGGTGGTAACTTCCAGGGTACCCCTATTGGAGTTTCTATGGACAATACAA
GGTTGGCTATTGCAGCCATTGGAAAGCTCATGTTTGCCCAATTTTCGGAGCTTGTGAATGATTTTTATA
ACAATGGGCTGCCATCTAATCTATCCGGAGGACGTAATCCAAGTTTGGATTATGGTTTCAAGGGTGCT
GAAATTGCTATGGCTTCTTACTGCTCTGAGCTGCAATTTTTGGGCAATCCAGTCACCAACCATGTCCAA
AGTGCTGAGCAACATAATCAGGACGTGAACTCCCTGGGTTTAATCTCCTCGAGGAAAACAGCAGAAG
CTGTTGAAATCTTGAAGCTCATGTCTACCACATTTTTGGTAGGCCTCTGCCAAGCTGTAGACCTGAGGC
ATCTGGAGGAGAACTTGAAGAGCACTGTGAAGAACACTGTAAGCCAAGTAGCCAAGAAAGTCCTAA
CTATGGGTGTCAACGGTGAGCTTCATCCTTCAAGATTTTGCGAGAAGGATTTGCTTAGAGTTGTTGACC
GAGAGTACATTTTTGCATACATTGACGACCCCTGCAGTGCAACCTACCCATTGATGCAAAAACTAAGA
CAAGTACTAGTTGAACACGCACTGAAGAATGGCGAAACTGAGAAGAACTTGAGCACTTCAATCTTCC
AAAAGATTGCAGCTTTTGAAGATGAATTGAAGACCCTTTTGCCAAAGGAGGTTGAAAGTGCTAGGGCT
GTGCTTGAGAGTGGAAATCCTGCCATTCCTAACAGAATTAAGGAGTGCAGGTCTTATCCATTGTACAA
GTTTGTAAGGGAAGAGTTGGGAACAGTTTATTTAACCGGTGAGAAAGTGACATCGCCTGGAGAGGAG
TTTGACAAGGTGTTCACGGCAATGTGCAAAGGACAGATCATTGATCCTCTGATGGAGTGTCTACAGAG
CTGGAATGGAGCTCCTCTACCAATTTCTTAG

Table S2. Ct values of 11 candidate genes of *A. dahurica*.

Treatments	Replicates	*SAND*	*PTBP*	*GAPDH*	*ACT*	*TIP41*	*CYP2*	*EF1α*	*UBC9*	*TUB6*	*YLS8*	*TUBA*
control (Sterile water)	1	26.7796	24.9754	20.5092	21.7106	25.2387	23.4786	18.5456	21.8313	26.9064	21.8604	18.9995
		26.0154	26.2328	21.3897	21.7227	24.7191	23.247	18.3417	21.6811	27.0457	21.6851	19.9748
		25.6375	25.3116	20.5816	21.5386	24.4656	23.5801	18.37	21.6032	26.7851	21.5499	19.4524
	2	24.7305	25.2539	20.5479	21.3273	25.2951	23.7765	18.58	22.0090	26.4068	21.7510	18.7972
		24.6274	25.3785	20.4767	21.4485	24.4048	23.9245	18.461	21.8286	27.0241	22.2643	18.8000
		24.5452	25.1263	20.6160	21.6452	24.3100	23.7071	18.5504	21.7288	26.5058	22.0746	18.6702
	3	25.2893	25.3798	20.3741	21.5655	24.8711	23.5564	18.5320	21.6482	26.7834	21.7418	19.2246
		25.4773	25.2239	20.3689	21.4896	24.7389	23.6190	18.4750	21.7803	26.7790	21.8642	19.1157
		25.4013	25.3357	21.3177	21.6414	24.6067	23.6816	18.4180	21.9154	26.7746	21.9866	19.0068
SA	1	21.7174	22.0633	18.6099	19.1928	22.7132	22.5562	16.5078	19.5212	25.6771	18.2956	17.8476
		21.9887	22.2833	18.4669	18.6875	22.976	22.2217	16.2551	19.4069	25.3198	18.0057	18.3292
		22.0935	22.3328	18.6546	19.5741	22.4223	22.9421	16.8064	19.0596	25.661	18.5891	17.6858
	2	22.0703	22.1887	18.5610	19.0431	22.3472	22.6054	16.4208	19.3074	26.1895	18.2997	18.5005
		21.9299	22.5842	18.8041	19.0439	22.0014	22.217	16.1815	19.2369	25.5632	18.1296	17.8355
		21.6310	22.6219	18.4918	18.8719	22.0394	22.0648	16.1237	19.1375	25.7548	18.1235	18.3066
	3	21.7614	22.2724	19.1491	18.7479	22.9699	22.6789	16.1549	19.163	26.0422	18.0615	18.1982
		21.9723	22.6694	18.4285	19.0275	22.0327	22.1581	16.3631	19.3403	25.4211	18.1858	17.7485
		22.3478	22.4948	18.3379	19.3941	22.4056	22.1642	16.105	19.1987	25.8507	18.0646	18.5728
NaCl	1	28.1626	27.6881	24.4006	23.8432	28.2176	27.2359	21.7818	23.3076	31.4448	22.6736	22.2059
		26.7949	26.3821	23.0588	24.0683	28.4427	26.1603	21.4205	23.2034	32.0233	22.1394	21.4259
		28.3305	26.7041	23.0741	24.5837	27.2681	26.4281	21.9248	23.1291	30.1621	22.1998	20.7281

	2	26.9465	26.6733	23.2006	23.5006	27.1249	26.6582	21.6848	23.0029	30.1340	22.4139	20.8482
		26.1030	26.8729	23.0847	23.7186	27.0179	25.9805	21.6646	23.2386	29.7338	22.3048	21.0040
		26.7713	27.5248	24.3431	24.8678	28.0863	27.4021	22.1602	23.5704	30.2343	23.3645	21.7983
	3	26.6052	27.0890	23.2472	24.5346	27.1836	26.0972	22.1598	23.0597	31.9393	22.4572	20.9445
		28.0183	26.7022	23.2406	23.6792	27.1727	26.6678	22.0168	23.0690	30.7128	22.4195	20.4672
		26.5768	26.8850	23.3479	24.0502	27.1328	26.0810	21.9005	22.9463	30.8291	22.3408	20.5944
MeJA	1	24.3847	22.8734	18.3225	18.8426	22.2894	22.3278	16.8268	19.3080	25.2183	18.6305	18.0991
		22.9342	22.2495	18.2781	19.2405	23.0147	22.0847	16.9592	19.2496	24.0228	18.2840	18.0059
		22.5406	22.4879	18.9565	19.7952	22.8795	22.2989	17.0654	19.1088	25.5384	18.4930	18.5912
	2	22.4508	22.4930	18.5868	20.0964	22.2652	22.364	16.8964	19.2758	26.0537	18.4089	18.0945
		22.6849	22.3192	18.4791	19.0890	22.3308	22.2729	16.6704	19.1972	25.9867	18.2951	17.7949
		22.5326	22.3650	18.6733	19.1450	22.4726	22.1747	17.0311	19.3972	26.0454	18.8597	17.7558
	3	22.4611	22.3332	18.6051	19.4749	22.6395	22.489	17.0059	19.2645	25.4616	18.5307	17.9584
		22.8289	22.4818	18.8528	20.1567	22.4409	22.357	16.9658	19.3586	25.6906	18.4776	18.1372
		23.0577	22.5166	18.7774	19.5848	22.3452	22.4686	17.0911	19.4295	25.6976	18.7601	17.6551
$CuSO_4$	1	24.0607	22.6570	18.8603	19.9596	22.7376	22.9057	17.5603	19.1686	27.276	18.1021	19.0073
		22.7708	22.4717	19.0393	20.1678	22.6403	23.4852	17.7866	19.1898	27.6375	17.9916	18.8502
		22.8999	22.7428	18.9328	20.1775	23.0452	23.0228	17.4818	19.2384	27.4543	18.069	19.0701
	2	22.7396	22.8036	18.9603	20.4849	22.6968	22.7883	17.7583	19.155	26.9114	18.0533	18.9489
		23.0127	22.6337	19.0128	20.3243	23.0193	22.9378	17.9536	19.2114	26.9023	18.1965	19.1975
		22.6950	22.4650	19.0761	19.9690	23.1276	23.0891	17.8238	19.2117	27.1723	18.4890	19.1018
	3	22.9718	22.9853	19.0278	18.9623	22.6176	23.2046	17.8569	19.1174	26.2809	18.0823	19.0054
		22.8171	22.4113	19.0395	20.2295	22.5242	22.8805	17.5526	19.1426	26.3443	18.4341	18.792
		23.0119	22.8497	19.3903	22.1814	22.6575	23.1692	17.6109	19.1524	26.9404	18.4422	18.5365

ETH	1	24.0966	20.6716	19.4353	18.9626	22.1293	20.0265	15.3937	19.1778	22.5001	18.1724	17.5492
		22.2369	20.4885	19.3298	18.9295	22.202	20.1662	15.6543	19.1699	21.9569	18.0848	17.417
		22.2467	20.7675	19.4226	18.9295	22.3121	19.9637	15.6087	19.4549	22.2923	18.1503	17.567
	2	22.2198	20.4324	19.5191	18.5836	22.0438	20.0572	15.6818	19.1448	21.8028	18.153	17.6431
		22.1774	20.4908	19.3958	19.0139	22.198	20.0246	15.7348	19.2686	22.0942	18.1609	17.5624
		22.0925	20.6835	19.7655	18.96	22.1717	20.0511	15.5777	19.2127	22.0105	18.3093	17.7466
	3	22.1983	20.6159	19.5304	18.928	22.2718	19.9943	15.5919	19.1625	22.2176	18.1849	17.8276
		22.2761	20.6559	19.6523	19.038	22.4386	20.087	15.7288	19.2283	22.3136	18.3114	17.7013
		22.4342	20.7286	19.4575	19.0204	22.1388	19.8966	15.6619	19.1548	22.0905	18.2378	17.6076
ABA	1	24.0077	22.5018	18.6524	19.1425	22.4655	22.1150	17.0420	19.7961	25.9089	18.3758	18.1349
		22.5823	22.3677	18.3390	18.1592	22.3470	22.2609	16.9196	19.2394	25.3115	18.3895	18.4216
		22.503	22.4611	18.6017	17.8520	22.1654	22.1051	16.9700	19.6825	25.3457	18.4803	17.6571
	2	22.5648	22.4203	19.2503	19.1916	22.2034	22.3039	16.7970	19.5989	26.5266	18.5078	17.6909
		22.2935	22.3245	18.9597	19.0963	22.5591	22.5924	16.9992	19.2933	25.8303	18.2199	17.7087
		22.5785	22.3098	18.5620	18.8621	22.2503	21.4463	17.0097	19.2701	25.5646	18.3540	17.6348
	3	22.3953	22.2074	18.5930	19.144	22.8901	22.4471	16.9291	19.3318	25.8438	18.4100	18.0863
		22.5240	22.4512	18.6340	19.2493	22.4294	22.3467	17.0162	19.3795	25.4276	18.4294	17.9449
		22.5337	22.3306	18.9883	19.8925	22.4138	23.0516	17.0604	19.5821	26.6910	19.1248	18.1537
mannitol	1	23.6581	21.8339	18.5137	19.0340	23.0985	21.1474	17.1646	20.2980	25.3128	18.3299	18.3832
		23.0597	21.4194	18.1239	18.2811	22.6946	21.1759	17.0115	19.1138	24.1675	18.6536	17.6903
		21.7865	21.3175	18.0425	18.7388	21.8179	21.0569	16.7867	20.4627	24.1910	18.6319	17.6852
	2	22.1652	21.8928	17.9240	18.1075	22.0898	21.4011	16.538	19.4848	24.1948	19.0621	18.1225
		21.2043	21.7542	18.4471	18.4762	21.6968	21.1170	16.9242	19.1340	24.6625	18.7812	17.7654
		22.2585	21.3455	18.5044	18.5512	22.7588	21.2373	17.1875	19.1273	25.0264	19.1060	18.1050

	3	21.3127	21.3485	18.3480	18.3805	21.7800	21.0833	17.2081	18.9498	24.3364	18.6725	17.5479
		22.2673	21.6331	18.4876	18.8006	21.7237	21.1166	17.0849	19.2776	24.5377	18.9303	17.7540
		21.2497	21.6899	18.4707	18.8202	22.2075	21.2919	17.0132	19.1814	24.4406	18.7935	17.7887

Table S3. Pairwise variation ($V_{n/n+1}$) analysis of 11 candidate reference genes calculated using geNorm.

	control	ETH	ABA	$CuSO_4$	mannitol	SA	NaCl	MeJA	total
$V_{2/3}$	0.068	0.061	0.134	0.162	0.126	0.088	0.159	0.127	0.179
$V_{3/4}$	0.054	0.064	0.098	0.113	0.097	0.091	0.117	0.094	0.131
$V_{4/5}$	0.054	0.104	0.099	0.154	0.086	0.077	0.107	0.075	0.117
$V_{5/6}$	0.052	0.077	0.088	0.124	0.078	0.082	0.101	0.076	0.115
$V_{6/7}$	0.056	0.106	0.078	0.097	0.065	0.096	0.083	0.080	0.110
$V_{7/8}$	0.053	0.103	0.078	0.092	0.073	0.083	0.081	0.073	0.098
$V_{8/9}$	0.052	0.094	0.077	0.096	0.077	0.076	0.096	0.073	0.085
$V_{9/10}$	0.047	0.105	0.079	0.097	0.082	0.089	0.093	0.078	0.083
$V_{10/11}$	0.078	0.093	0.067	0.108	0.079	0.077	0.104	0.069	0.099

Table S4. Stability rank of 11 candidate genes by NormFinder.

Rank	control	ETH	ABA	$CuSO_4$	mannitol	SA	NaCl	MeJA	total
1	*ACT*	*EF1α*	*UBC9*	*GAPDH*	*GAPDH*	*EF1α*	*ACT*	*GAPDH*	*TIP41*
	0.01	0.015	0.123	0.150	0.153	0.095	0.218	0.101	0.258
2	*EF1α*	*ACT*	*GAPDH*	*TIP41*	*TUB6*	*UBC9*	*GAPDH*	*UBC9*	*ACT*
	0.075	0.070	0.145	0.201	0.171	0.126	0.22	0.172	0.277
3	*UBC9*	*UBC9*	*TIP41*	*EF1α*	*CYP2*	*ACT*	*TUBA*	*ACT*	*EF1α*
	0.091	0.145	0.213	0.319	0.182	0.135	0.271	0.217	0.317
4	*TUB6*	*TIP41*	*EF1α*	*UBC9*	*ACT*	*GAPDH*	*TIP41*	*EF1α*	*UBC9*
	0.127	0.218	0.252	0.376	0.205	0.149	0.3	0.218	0.325
5	*GAPDH*	*CYP2*	*ACT*	*CYP2*	*TIP41*	*TIP41*	*CYP2*	*TIP41*	*GAPDH*
	0.175	0.260	0.316	0.424	0.254	0.242	0.302	0.241	0.389
6	*YLS8*	*YLS8*	*PTBP*	*ACT*	*UBC9*	*PTBP*	*PTBP*	*CYP2*	*CYP2*
	0.2	0.278	0.382	0.431	0.284	0.343	0.346	0.332	0.412
7	*CYP2*	*SAND*	*CYP2*	*PTBP*	*YLS8*	*CYP2*	*UBC9*	*PTBP*	*PTBP*
	0.208	0.468	0.383	0.454	0.324	0.444	0.375	0.360	0.468
8	*PTBP*	*TUBA*	*TUBA*	*SAND*	*EF1α*	*TUB6*	*EF1α*	*TUBA*	*SAND*
	0.253	0.596	0.420	0.504	0.425	0.480	0.435	0.427	0.539
9	*TUBA*	*TUB6*	*TUB6*	*TUBA*	*TUBA*	*TUBA*	*SAND*	*TUB6*	*TUBA*
	0.311	0.636	0.491	0.577	0.523	0.544	0.583	0.458	0.542
10	*TIP41*	*PTBP*	*SAND*	*TUB6*	*PTBP*	*YLS8*	*YLS8*	*YLS8*	*YLS8*
	0.312	0.697	0.497	0.728	0.528	0.561	0.689	0.513	0.580
11	*SAND*	*GAPDH*	*YLS8*	*YLS8*	*SAND*	*SAND*	*TUB6*	*SAND*	*TUB6*
	0.654	0.699	0.518	0.810	0.597	0.579	0.786	0.516	0.743

Table S5. Stability rank of 11 candidate genes by Bestkeeper.

Rank	control	ETH	ABA	$CuSO_4$	mannitol	SA	NaCl	MeJA	total
1	*EF1α*	*GAPDH*	*TUB6*	*TUBA*	*TUBA*	*TUBA*	*YLS8*	*TUBA*	*TUBA*
CV±SD	0.45±0.08	2.85±0.57	2.15±0.56	1.21±0.23	3.26±0.60	2.64±0.49	1.42±0.32	2.98±0.55	3.94±0.74
2	*UBC9*	*TUBA*	*TUBA*	*TUB6*	*EF1α*	*TUB6*	*UBC9*	*TUB6*	*CTP2*
CV±SD	0.50±0.11	3.93±0.72	3.08±0.57	1.10±0.29	4.05±0.71	1.97±0.51	2.95±0.67	2.34±0.61	5.00±1.15
3	*ACT*	*TIP41*	*CYP2*	*CYP2*	*TUB6*	*CYP2*	*PTBP*	*CYP2*	*EF1α*
CV±SD	0.59±0.13	5.22±1.21	2.91±0.66	1.30±0.30	4.22±1.07	2.59±0.59	2.86±0.75	2.74±0.63	6.46±1.16
4	*CYP2*	*UBC9*	*EF1α*	*EF1α*	*UBC9*	*GAPDH*	*SAND*	*EF1α*	*GAPDH*
CV±SD	0.78±0.18	6.07±1.23	4.11±0.72	2.04±0.37	5.55±1.13	5.12±1.00	3.53±0.93	4.18±0.73	5.94±1.18
5	*YLS8*	*ACT*	*GAPDH*	*GAPDH*	*GAPDH*	*EF1α*	*TUBA*	*GAPDH*	*TUB6*
CV±SD	0.93±0.20	6.33±1.27	4.81±0.94	1.02±0.79	5.90±1.14	6.01±1.03	4.72±0.96	5.12±0.99	5.01±1.32
6	*TUB6*	*EF1α*	*UBC9*	*ACT*	*CYP2*	*TIP41*	*ACT*	*ACT*	*UBC9*
CV±SD	0.80±0.22	8.16±1.37	5.45±1.11	3.88±0.81	5.28±1.17	4.74±1.11	5.26±1.21	4.90±1.00	6.58±1.36
7	*GAPDH*	*SAND*	*TIP41*	*TIP41*	*TIP41*	*ACT*	*GAPDH*	*TIP41*	*TIP41*
CV±SD	1.13±0.23	6.26±1.48	4.78±1.12	3.98±0.94	5.23±1.22	5.98±1.20	5.92±1.32	4.55±1.07	5.88±1.40
8	*PTBP*	*CYP2*	*ACT*	*SAND*	*ACT*	*UBC9*	*TIP41*	*UBC9*	*ACT*
CV±SD	1.12±0.28	8.03±1.72	6.27±1.25	4.85±1.16	7.26±1.43	5.96±1.21	5.05±1.33	5.90±1.20	6.94±1.43
9	*TIP41*	*YLS8*	*SAND*	*UBC9*	*YLS8*	*PTBP*	*CYP2*	*SAND*	*SAND*
CV±SD	1.42±0.35	8.95±1.76	5.65±1.34	6.18±1.25	7.41±1.48	6.08±1.44	5.50±1.39	5.33±1.27	6.66±1.60
10	*TUBA*	*TUB6*	*PTBP*	*PTBP*	*SAND*	*SAND*	*EF1α*	*PTBP*	*YLS8*
CV±SD	2.09±0.40	9.28±2.23	6.12±1.44	5.48±1.30	6.86±1.61	7.09±1.65	7.92±1.62	5.94±1.40	8.88±1.79
11	*SAND*	*PTBP*	*YLS8*	*YLS8*	*PTBP*	*YLS8*	*TUB6*	*YLS8*	*PTBP*
CV±SD	2.97±0.75	10.16±2.29	8.20±1.63	8.93±1.76	7.89±1.82	8.96±1.76	6.61±1.93	8.07±1.60	7.65±1.82

Table S6. Expression stability values of 11 candidate genes calculated using the RefFinder.

**Rank**	**control**	**SA**	**NaCl**	**MeJA**	**$CuSO_4$**	**ETH**	**ABA**	**mannitol**	**total**
1	*EF1α*	*EF1α*	*GAPDH*	*GAPDH*	*GAPDH*	*EF1α*	*UBC9*	*GAPDH*	*TIP41*
	1.565	1.495	1.934	1.495	2.115	1.565	1.565	1.495	1.627
2	*ACT*	*UBC9*	*ACT*	*ACT*	*TIP41*	*UBC9*	*GAPDH*	*TUB6*	*UBC9*
	1.968	2.378	2.632	2.913	3.027	2.449	2.783	1.861	2.913
3	*UBC9*	*ACT*	*CYP2*	*UBC9*	*UBC9*	*ACT*	*TIP41*	*CYP2*	*ACT*
	2.213	3.705	3.663	3.13	3.464	2.783	2.817	3.568	3.364
4	*TUB6*	*GAPDH*	*TUBA*	*EF1α*	*EF1α*	*TIP41*	*EF1α*	*ACT*	*EF1α*
	4.12	4	3.873	3.936	3.663	3.722	4	4.757	3.663
5	*CYP2*	*TUBA*	*TIP41*	*TUBA*	*PTBP*	*GAPDH*	*TUB6*	*TUBA*	*GAPDH*
	5.091	5.196	4.681	4.601	4.527	5.609	4.88	5.196	4.162
6	*YLS8*	*TIP41*	*PTBP*	*CYP2*	*CYP2*	*CYP2*	*CYP2*	*UBC9*	*CYP2*
	5.180	5.233	5.045	5.045	4.606	5.623	5.01	5.422	4.559
7	*GAPDH*	*TUB6*	*UBC9*	*TIP41*	*TUBA*	*TUBA*	*TUBA*	*EF1α*	*TUBA*
	5.916	5.657	5.118	5.144	5.196	5.657	5.264	5.657	5.335
8	*PTBP*	*CYP2*	*YLS8*	*TUB6*	*ACT*	*YLS8*	*ACT*	*TIP41*	*PTBP*
	7.737	5.664	5.623	6.000	6.481	6.64	6.325	5.916	7.837
9	*TUBA*	*PTBP*	*SAND*	*PTBP*	*TUB6*	*SAND*	*PTBP*	*YLS8*	*SAND*
	9.24	6.64	7.348	8.149	6.687	7	7.841	6.853	8.485
10	*TIP41*	*YLS8*	*EF1α*	*YLS8*	*SAND*	*TUB6*	*SAND*	*PTBP*	*TUB6*
	9.74	10.241	8.459	10.241	8	9.487	10.215	10.241	9.032
11	*SAND*	*SAND*	*TUB6*	*SAND*	*YLS8*	*PTBP*	*YLS8*	*SAND*	*YLS8*
	11	10.741	11	10.462	11	10.741	10.488	10.741	9.457
